# Polarisation Engineering in Covalent Organic Frameworks for Catalysis

**DOI:** 10.1007/s40820-026-02183-y

**Published:** 2026-04-28

**Authors:** Xinqiang Wang, Xiaoning Li, Minna Li, Zhixuan Li, Zhihao Lei, Shujuan Huang, Jiabao Yi, Zhong-Yong Yuan, Liqun Ye, Wen-Gang Cui, Tianyi Ma, Hongge Pan, Xinwei Guan

**Affiliations:** 1 Institute of Science and Technology for New Energy, Xi’an Technological University, Xi’an, 710021 People’s Republic of China; 2 Centre for Atomaterials and Nanomanufacturing (CAN), School of Science, RMIT University, Melbourne, VIC 3000 Australia; 3 Department of Applied Physics, Aalto University, 00076 Aalto, Espoo Finland; 4 Department of Chemical Engineering and Interdisciplinary Research Centre for Hydrogen Technologies and Carbon Management (IRC-HTCM), King Fahd University of Petroleum and Minerals, 31261 Dhahran, Saudi Arabia; 5 School of Engineering, Macquarie University, Sydney, NSW 2109 Australia; 6 Collaborative Innovation Center of Materials Science, Nankai University, Tianjin, 300350 People’s Republic of China; 7 College of Materials and Chemical Engineering, Key Laboratory of Inorganic Nonmetallic Crystalline and Energy Conversion Materials, China Three Gorges University, Yichang, 443002 People’s Republic of China

**Keywords:** Covalent organic frameworks, Polarisation engineering, Built-in electric field, Charge separation, Catalysis

## Abstract

Polarisation in covalent organic framework (COF) catalysts has emerged as an effective strategy to reduce strong excitonic binding and to improve charge transport through built-in electric fields. Unlike conventional inorganic ferroelectrics or polar materials, COFs enable programmable polarisation through molecular and lattice design, allowing internal fields to be tuned in strength and direction. In this review, we provide a comprehensive analysis of polarisation in COFs, from its physical origins to its functional roles in catalysis. We first examine the multiscale origins of polarisation in COFs, encompassing bond-level electronic asymmetry, conjugation-mediated propagation, and framework-level structural organisation that governs dipole alignment and cancellation. We then summarise how polarisation is characterised experimentally and theoretically across different electronic and catalytic states, including ground-state electrostatic potential landscapes, photoexcited-state charge dynamics, and polarisation effects at solid–liquid catalytic interfaces. Finally, through representative photocatalytic and electrocatalytic case studies, we illustrate how deliberate polarisation engineering reshapes charge separation, transport, and reaction pathways across diverse catalytic reactions, and conclude by discussing the key opportunities and challenges for translating polarisation into a predictive design principle for COFs. By connecting the origins, characterisations, regulating strategies, and catalytic mechanisms, this review provides a more integrated perspective on polarisation phenomena in next-generation COF catalysts.

## Highlights

- Establishes a unified framework linking the origins, descriptors, and catalytic functions of polarisation in covalent organic frameworks (COFs).
- Systematically categorises polarisation engineering strategies in COFs and correlates them with excitonic, electronic, and interfacial descriptors.
- Demonstrates how programmable internal electrostatics enable selective and efficient photocatalytic and electrocatalytic reactions.

## Graphical abstract

**Figure:**
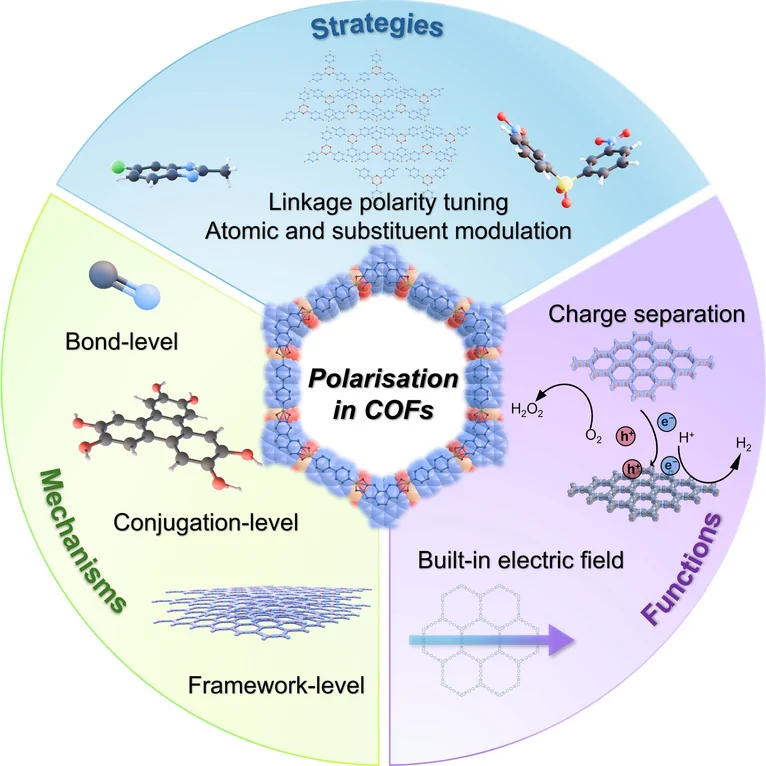

## Introduction

Covalent organic frameworks (COFs) are a distinctive family of crystalline porous materials constructed from organic building blocks linked into periodic networks through robust covalent linkages [1–6]. Structurally and chemically, COFs are characterised by several key features: (i) long-range crystallinity with well-defined and addressable topology [7, 8], (ii) high surface area with tunable and programmable pore environments [9, 10], and (iii) chemically tailorable π-skeletons [11, 12]. These intrinsic features collectively endow COFs with advantages that are particularly important for catalysis, including precise control over mass transport, the ability to tailor the local environment of active sites, and efficient charge separation and transport, making them competitive with or superior to many conventional porous materials [13–19]. Owing to these advantages, COFs have been widely investigated as platforms for photocatalysis [9, 20–25], electrocatalysis [26–33], and environmental remediation [18, 34–39].

Despite nearly two decades of development since their first report in 2005 [2]. COF-based catalysts still suffer from intrinsic electronic bottlenecks that fundamentally distinguish them from conventional inorganic semiconductors [40–42]. Many conjugated COFs exhibit low dielectric constants and pronounced excitonic effects, leading to tightly bound electron–hole pairs and inefficient free-carrier generation [43–45]. Charge transport is further hindered by imperfect crystallinity, grain boundaries, stacking disorder, and trap-rich interfaces, which collectively accelerate non-radiative recombination and restrict carrier diffusion lengths [1]. Consequently, even when a COF provides abundant accessible sites and favourable adsorption motifs, its catalytic turnover can remain constrained by inefficient charge separation, fast recombination, and/or limited charge transport to reactive interfaces, i.e., an inability to deliver charges with sufficient directionality, lifetime, and driving force [46]. To address these challenges, a range of strategies has been explored, including improving crystallinity, extending *π*-conjugation, introducing donor–acceptor (D–A) units, hybridising COFs with cocatalysts or conductive scaffolds, and optimising pore microenvironments [47]. Nevertheless, these approaches are often implemented on a case-by-case basis, and a transferable understanding of how the internal electrostatic landscape governs charge migration, accumulation, and interfacial chemistry has yet to be established.

**Fig. 1:**
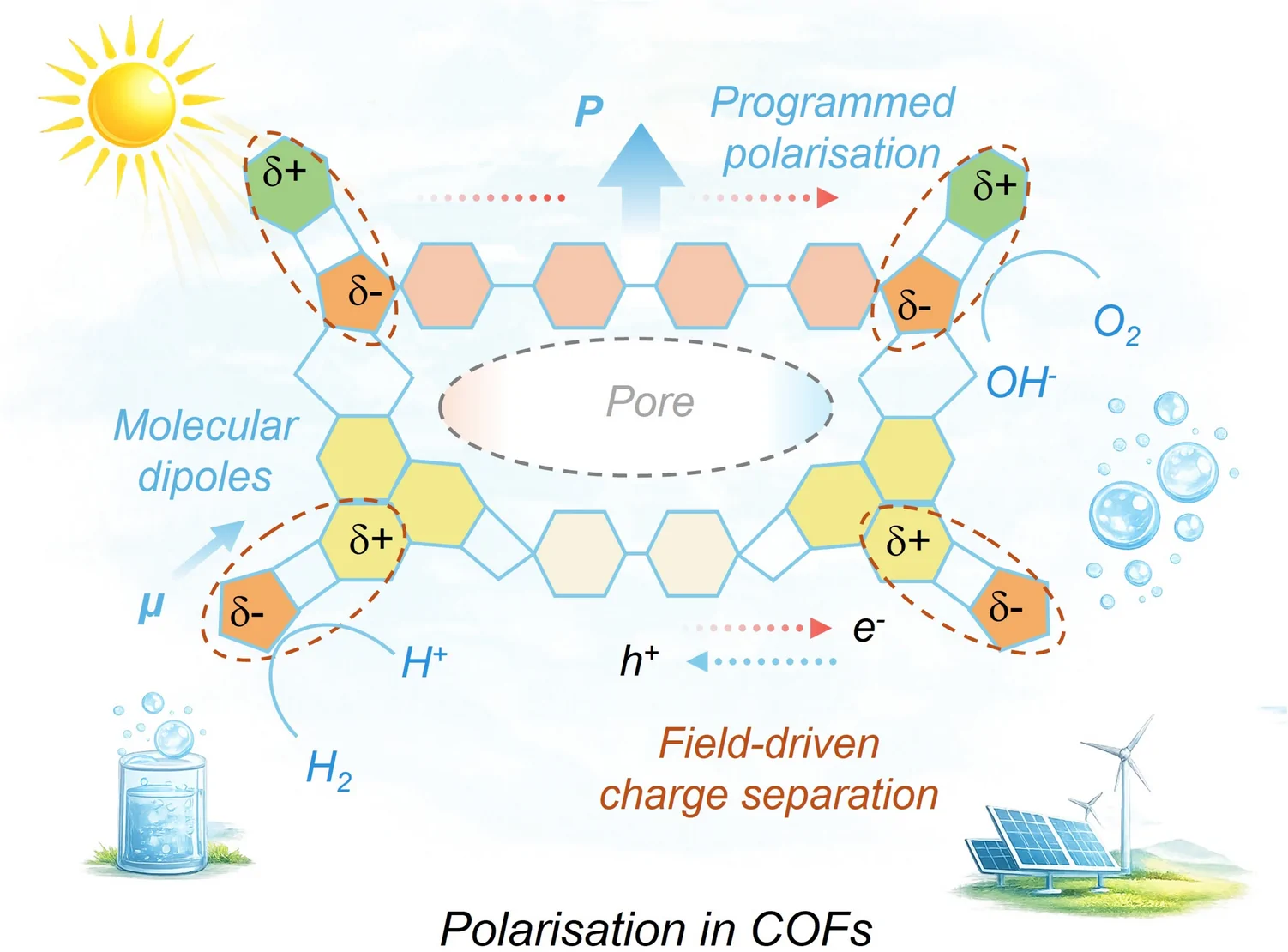
Schematic illustration of polarisation in COFs, where programmed alignment of molecular dipoles (*μ*) generates macroscopic polarisation (*P*) and built-in electric fields, enabling field-driven charge separation and modulation of catalytic adsorption at reactive sites

In this context, polarisation, which is an asymmetric distribution of charge density that generates built-in electric fields, offers a compelling and underexplored route to reprogram charge and reaction landscapes in COFs [48, 49]. While polarisation is well established in inorganic ferroelectrics and piezoelectrics, its implementation in COFs is conceptually distinct. In classical ferroelectrics, polarisation typically arises from collective ionic displacements within a non-centrosymmetric crystal lattice and is characterized by spontaneous, switchable macroscopic polarisation, often evidenced by polarisation-electric field hysteresis loops [48]. The magnitude and orientation of polarisation in such systems are largely dictated by bulk lattice symmetry and long-range ionic off-centring. In contrast, polarisation in COFs originates from fundamentally different structural and electronic principles. Rather than cooperative ionic displacement within an inorganic lattice, COFs predominantly originate from electronically programmed charge asymmetry embedded within polar covalent linkages, D-A motifs, or ionic functionalities distributed across a *π*-conjugated framework. Most reported COFs crystallise in centrosymmetric or weakly polar space groups and therefore do not exhibit switchable spontaneous polarisation characteristic of classical ferroelectrics. Instead, their built-in electric fields emerge from the spatial integration of molecular dipoles within a periodically connected lattice. Importantly, the reticular and modular nature of COFs enables polarisation to be chemically encoded at multiple hierarchical levels, allowing fine control over the magnitude, directionality, and spatial distribution of internal electrostatic potential landscapes (Fig. 1) [50]. Beyond merely suppressing recombination, polarisation in COFs can lower exciton binding energy, promote directional carrier drift and spatial redox separation, and reshape band bending and local work functions at interfaces [51, 52]. The molecular and porous architecture further allows these built-in fields to be coupled directly with specific reaction microenvironments, offering an electrostatic tunability that is not readily accessible in conventional inorganic systems [53, 54]. For example, in oxygen reduction reactions, even modest changes in built-in field strength can alter the relative stability of *OOH versus *O/*OH, thereby favouring the two-electron H_2_O_2_ pathway over O–O bond cleavage [55, 56]. This emphasises an important point from recent studies: polarisation does not follow a simple stronger-is-better trend but must be tuned in both strength and spatial distribution to the target reaction coordinate and interfacial energetics.

**Fig. 2:**
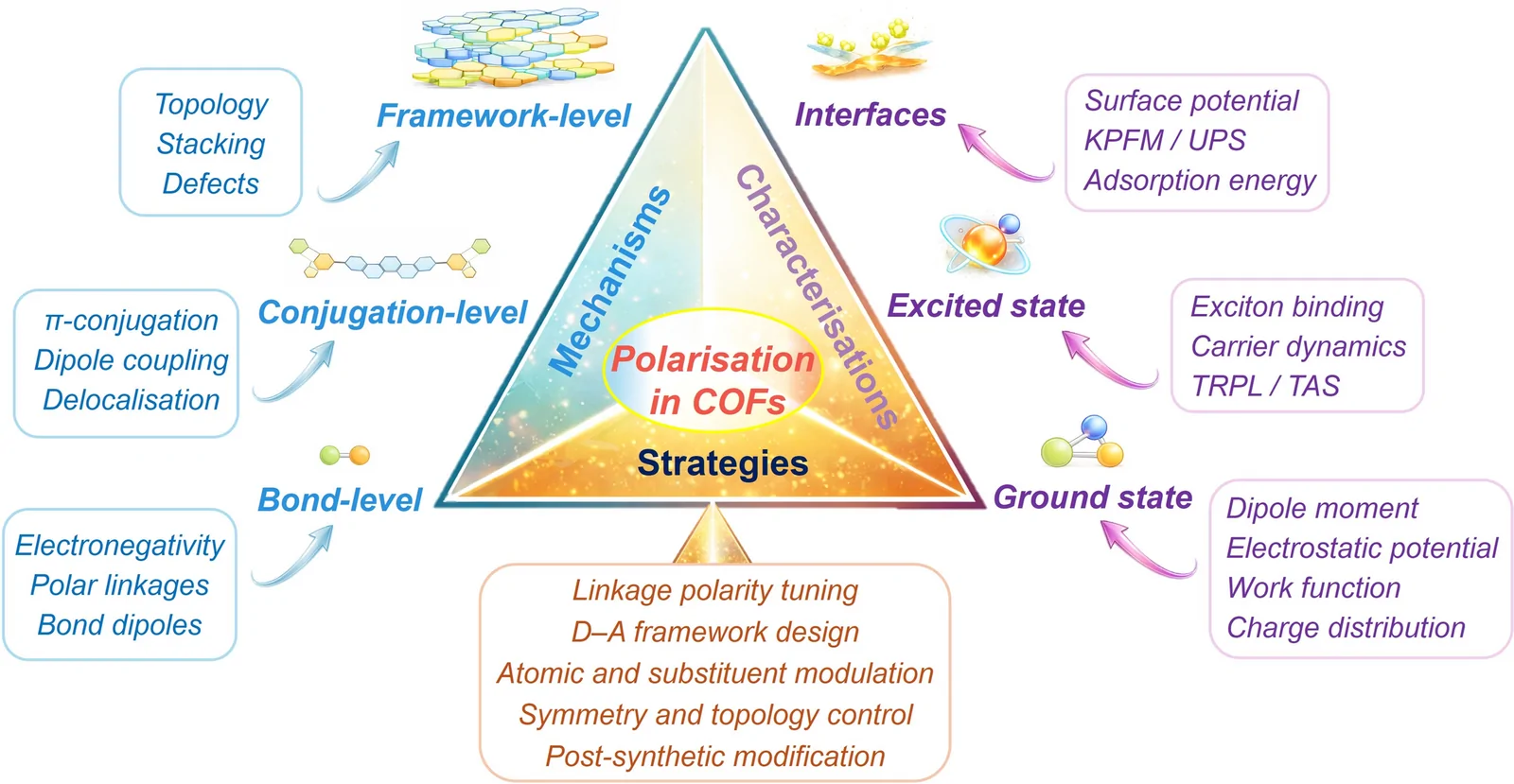
Conceptual framework illustrating the hierarchical origins of polarisation in COFs, together with representative polarisation engineering strategies and experimental and computational characterisation tools

From the discussion above, it is clear that a more unified framework is desired to understand and exploit polarisation in COFs. Unfortunately, most existing COF reviews focus on single or a few aspects like linkage chemistry, crystallinity, porosity, and D-A electronic design, while polarisation-related concepts remain dispersed across individual photocatalytic and electrocatalytic studies with inconsistent metrics and terminology [57–62]. This review aims to fill this gap by organising polarisation engineering in COFs from its fundamental origins to its catalytic applications (Fig. 2). We first examine the multiscale origins of polarisation in COFs, encompassing bond-level electronic asymmetry arising from polar covalent linkages, conjugation-mediated propagation of local dipoles along the *π*-skeleton, and framework-level structural organisation that governs dipole alignment, cancellation, and collective expression through symmetry, topology, stacking, torsion, and defects. We then summarise how polarisation is experimentally and theoretically characterised across different electronic and catalytic states, from ground-state electrostatic descriptors such as dipole moments and electrostatic potential distributions, to photoexcited-state signatures including exciton binding energies and carrier lifetimes, as well as interface-specific probes that capture polarisation under electrochemical screening and liquid-phase conditions. Building on this physical picture, we categorise the major polarisation-engineering strategies developed in COFs, including linkage polarity tuning, D–A framework engineering, atomic and substituent modulation, symmetry and topology control, and post-synthetic modification via ionic, covalent, or defect-based routes. In this review, polarisation engineering refers specifically to strategies that generate intrinsic electrostatic asymmetry within a single periodically connected COF lattice. D–A design and defect modulation are considered within this scope only when they induce measurable intraframework dipoles or internal electric field gradients, rather than merely tuning band structures or introducing additional active sites. In addition, conventional heterojunctions formed by physically distinct phases fall outside this definition unless the electrostatic effect is structurally encoded within the COF lattice itself. These case studies translate the concepts into practical, transferable guidelines for tuning polarisation in COFs to improve charge dynamics and steer reaction pathways in specific catalytic applications. Finally, we discuss emerging opportunities for polarised COFs in electrochemical energy storage, including rechargeable batteries and metal-air systems, and outline key challenges for establishing polarisation engineering as a general paradigm for next-generation COF-based catalysts and devices.

## Fundamentals of Polarisation in COFs

### Origins and Mechanisms of Polarisation

**Fig. 3:**
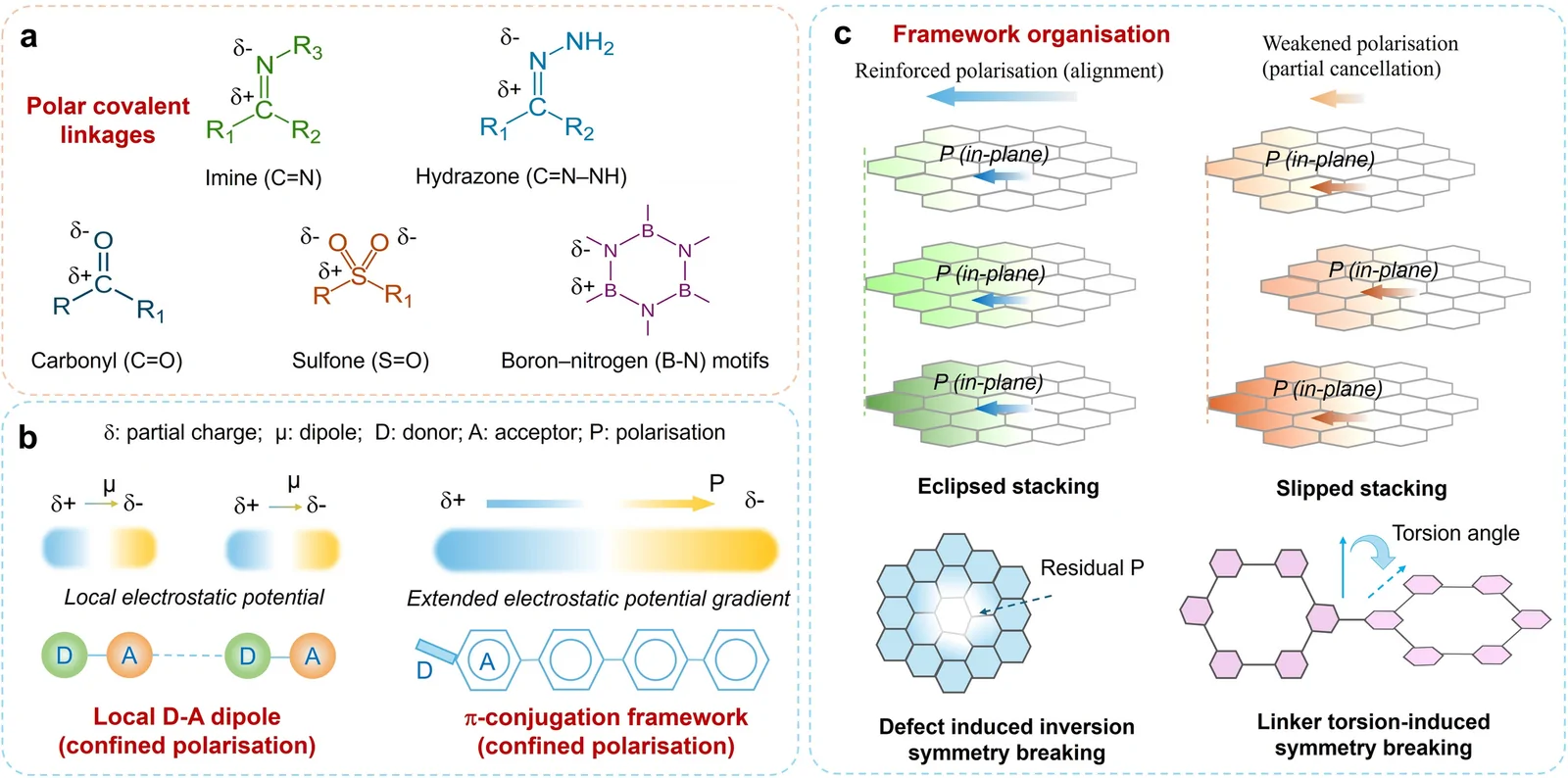
**a** Representative polar covalent linkages introducing local electronic asymmetry, where δ⁺ and δ⁻ denote partial atomic charges. **b** Comparison between locally polarised and π-conjugated polar frameworks. **c** Representative framework organisation effects, where eclipsed stacking reinforces dipole alignment, slipped stacking weakens net polarisation through partial cancellation, and defects or linker torsion locally break inversion symmetry, generating residual local polarisation

Generally, polarisation in COFs is best understood as an emergent electrostatic phenomenon arising from charge asymmetry encoded at the bond and motif levels and then amplified, cancelled, or reoriented by framework connectivity, lattice symmetry, stacking order, and post-synthetic reprogramming. In contrast to classical inorganic ferroelectrics, where polarisation is often associated with bulk lattice distortions and switchable dipoles, COF polarisation is typically non-ferroelectric and originates from programmable molecular-scale charge redistribution. Importantly, the functional consequence is not limited to a single net dipole moment. Depending on symmetry and topology, a COF can exhibit dipolar, quadrupolar, or higher-order multipolar charge distributions, each producing distinct electrostatic potential landscapes that regulate exciton behaviour, carrier migration, and interfacial reaction energetics. Conceptually, its origin can be classified into three interconnected levels (Fig. 3): (i) bond-level electronic asymmetry, arising from polar covalent linkages and local coordination environments, generating intrinsic bond-resolved dipoles and local electrostatic contrast; (ii) conjugation-mediated electronic coupling, through which local asymmetry is propagated, integrated, or reshaped across the extended *π* network, enabling discrete bond dipoles or motif-scale donor–acceptor contrast to evolve into continuous electrostatic potential gradients and built-in internal fields; and (iii) framework-level structural organisation, encompassing lattice symmetry, topology, stacking order, and orientational registry, which governs whether local dipoles coherently align, partially cancel, or reorganise into dipolar or multipolar charge distributions that ultimately determine the spatial form and functional expression of polarisation.

#### Bond-Level Electronic Asymmetry

At the most fundamental level, local polarisation in COFs arises from bond-level electronic asymmetry associated with polar covalent linkages. Differences in electronegativity drive an uneven charge distribution along the bond axis, giving rise to intrinsic, bond-resolved dipoles [63]. Widely used COF linkages, as shown in Fig. 3a, including imine (C=N) [64], hydrazone (C=N–NH) [65], carbonyl (C=O) [66], sulfone (S=O) [67], and boron–nitrogen motifs (B–N) [68] inherently possess bond-resolved dipoles due to pronounced electronegativity differences between the bonded atoms. These bond dipoles constitute the elementary source of charge asymmetry in COFs and are intrinsically local in nature. Their magnitude is primarily governed by electronegativity contrast, bond polarity, and resonance within the linkage itself and is therefore relatively insensitive to higher-order framework features such as lattice symmetry or long-range periodicity. For example, imine (C=N) and hydrazone (C=N–NH) linkages represent the most widely used class of COF connections and exhibit intermediate polarity, and their bond dipoles originate from both electronegativity differences and resonance-assisted charge redistribution along the conjugated backbone.

Importantly, the presence of a polar bond does not automatically imply the existence of a functional built-in electric field. A bond dipole is a local quantity, whereas catalytic consequences depend on how such dipoles are subsequently integrated, oriented, and coupled within the extended framework. This distinction explains why COFs constructed from only moderately polar linkages can still exhibit pronounced electrostatic potential contrast at the framework level, while materials containing highly polar bonds may show little net polarisation if symmetry or connectivity enforces cancellation. Thus, bond-level electronic asymmetry defines the origin of polarisation, but not its eventual functional manifestation.

#### Conjugation-Mediated Electronic Coupling

Once local bond-level asymmetry is embedded in a periodic and *π*-conjugated lattice, polarisation can extend beyond individual linkages and emerge as a collective electronic feature. A key point is that *π*-conjugation plays a central role in converting local electronic asymmetry into long-range polarisation in COFs [69–71]. When linkages are embedded within extended *π*-conjugated backbones, individual dipoles no longer behave as isolated entities, as demonstrated in Fig. 3b. Instead, electronically coupling mediated by *π*-conjugation enables charge redistribution to propagate across the framework, giving rise to delocalised charge deformation and the emergence of spatially continuous electrostatic potential (ESP) gradients. Importantly, this process does not create polarity but determines how efficiently pre-existing local dipoles are electronically coupled and spatially expressed within the lattice. Through this coupling, molecular-scale asymmetry is transformed into intrinsic electric fields embedded within the COF lattice. Such built-in fields bias carrier motion, promote spatial separation of photoexcited electrons and holes, and partially screen Coulomb attraction. As a result, exciton binding energies can be reduced and long-range charge transport facilitated, even in systems where the intrinsic polarity of individual linkages is modest. From this perspective, conjugation-mediated electronic coupling is the key mechanism by which local dipoles are electronically integrated into a coherent polarisation, providing the microscopic origin of the built-in electric fields in polarised COFs. The magnitude and directionality of this polarisation, however, remain subject to subsequent regulation by framework-level structural organisation, as discussed in the following section.

#### Framework-Level Structural Organisation

While conjugation-mediated coupling integrates local dipoles electronically, framework-level structural organisation ultimately determines whether these dipoles cancel, align, or cooperatively amplify into net polarisation within COFs [72]. The periodic and modular nature of the lattice provides multiple structural degrees of freedom that directly control dipole alignment [73]. For example, lattice symmetry and topology dictate whether polar units are in centrosymmetric motifs, leading to dipole cancellation, or in non-centrosymmetric configurations that allow dipoles to add constructively (Fig. 3c). Stacking geometry further modulates this effect in layered COFs. Eclipsed stacking can reinforce dipole alignment across layers, whereas slipped or offset stacking may either weaken or redirect the net polarisation depending on the relative orientation of polar motifs. It is noteworthy that framework organisation does not introduce new bond-level polarity, but governs the collective outcome of pre-existing electronic asymmetry under periodic boundary conditions. In addition, structural features such as linker torsion, asymmetric D–A connectivity, and defect-induced local asymmetry can locally break inversion symmetry, preventing complete dipole cancellation even when the ideal lattice would otherwise be centrosymmetric.

In typical two-dimensional COFs, in contrast to isolated molecular dipoles that may cancel locally, cooperative alignment of dipoles over extended length scales can generate collective polarisation domains spanning entire framework sheets [74, 75]. Depending on the organisation of these polar motifs, polarisation may manifest predominantly as in-plane or out-of-plane fields, or as multipolar charge distributions, each exerting distinct influences on charge delocalisation, transport anisotropy, and interfacial reactivity. In essence, polarisation in COFs should be understood as a multiscale electronic phenomenon jointly encoded by covalent bonding and framework architecture. This hierarchical origin distinguishes COF polarisation from that of conventional inorganic ferroelectrics, in which polarisation is primarily determined by bulk lattice distortion. As will be demonstrated in the following sections, this structurally programmable polarisation provides the physical foundation for directional charge transport, interfacial electrostatic modulation, and catalytic functionality in polarised COFs.

### Quantifying Polarisation in COFs

Polarisation in COFs arises from subtle electronic asymmetry embedded in molecular linkages and *π*-conjugated frameworks, rather than from large-scale lattice distortions. As a consequence, polarisation cannot be adequately described by a single quantity; it is more appropriately quantified using a series of descriptors that span ground-state electronic asymmetry, excitonic properties, carrier dynamics, and interfacial electrostatic responses, thereby forming the basis for establishing structure–property relationships in polarised COFs. In this section, we outline the key polarisation-related descriptors most relevant to the systems and clarify their physical significance. To avoid ambiguity, it is necessary to distinguish several closely related terms. The dipole moment (*μ*) is defined as:

1 $$\mu =\sum_{i}{q}_{i}{r}_{i}$$

where ${q}_{i}$ and ${r}_{i}$ denote the charge and position of each constituent, and it describes charge asymmetry at the molecular or fragment level. At the framework scale, dipole density corresponds to the dipole moment normalised by volume (or area in layered systems), which is equivalent to the macroscopic polarisation vector in a periodic lattice:

2 $$P=\frac{\mu }{V}$$

where *P* denotes the dipole density (i.e., polarisation density), *μ* is the electric dipole moment defined above, *V* is the volume of the molecular unit, repeating cell, or material domain considered. By contrast, polarity density is used here to characterise the spatial distribution and gradient of electrostatic asymmetry, particularly at surfaces or interfaces, where local potential variations rather than net dipole magnitude dominate functional behaviour. Unlike dipole density (polarisation), which represents the dipole moment per unit volume and corresponds to a bulk vector quantity, polarity density reflects local variations in electrostatic potential or charge distribution, typically quantified via the electrostatic potential (ESP) profile or its spatial gradient.

#### Ground-State Polarisation Descriptors

Ground-state descriptors capture the intrinsic electronic asymmetry encoded in the static structure of a COF prior to external excitation. At this level, polarisation originates from the arrangement of polar linkages, D–A backbones, substituents, defects, or ionic motifs within the π-conjugated framework, defining the baseline internal electric fields experienced by charge carriers. The most direct quantitative descriptor of ground-state polarisation is the dipole moment, commonly normalised as a dipole density per unit area or volume to enable comparison across extended frameworks. Dipole-based metrics quantify the overall magnitude and direction of charge separation induced by framework asymmetry. Even in structures where global dipoles partially cancel due to symmetry, local or domain-resolved dipole components remain highly informative for assessing polarisation tendencies at the molecular and sublattice levels.

ESP distributions, derived from the same ground-state charge density, offer a complementary, spatially resolved view of polarisation [76]. ESP maps visualise regions of electron accumulation and depletion and reveal how local bond dipoles couple through *π*-conjugated networks to generate internal potential gradients. Such potential differences enable intuitive comparison of polarisation strength and directionality across isostructural COF series. Together, dipole-related descriptors quantify the magnitude and orientation of polarisation, while ESP analyses elucidate its spatial distribution, jointly defining the intrinsic electronic origin and internal electrostatic landscape of COF lattices.

#### Excited-State Polarisation Descriptors

While ground-state descriptors establish the existence and magnitude of intrinsic polarisation, its functional consequences emerge most clearly at the excitonic and carrier-dynamic levels under external excitation. Upon photoexcitation or charge injection, the built-in polarisation modulates electron–hole interactions, charge-separation pathways, and carrier migration within the framework. A central descriptor at this stage is the exciton binding energy (*E*_*b*_), which reflects the Coulombic attraction between photoexcited electrons and holes. In polarised COFs, built-in electric fields and local dielectric responses can partially screen electron–hole interactions, thereby reducing *E*_*b*_ and facilitating exciton dissociation [77]. Hence, *E*_*b*_ provides a quantitative link between intrinsic electronic asymmetry and the generation of mobile charge carriers. However, *E*_*b*_ values are inherently sensitive to modelling assumptions and measurement conditions and should therefore be interpreted in conjunction with complementary excited-state descriptors [78, 79]. Beyond exciton energetics, excited-state charge-density redistribution offers real-space insight into polarisation-assisted charge separation. By visualising preferential localisation of electrons and holes on distinct structural motifs, such as donor–acceptor segments, polar linkages, or defect sites, these descriptors reveal how intrinsic polarisation biases charge-separation pathways within a single-component COF lattice.

Carrier lifetimes constitute another key descriptor of polarisation effects at the excited-state level [80]. Prolonged lifetimes and suppressed recombination rates are commonly associated with strengthened internal fields and more efficient spatial separation of charge carriers, whereas shortened emissive lifetimes may indicate rapid field-assisted extraction to interfaces. Together, excitonic and carrier-level descriptors, including *E*_*b*_, charge-density redistribution, and carrier lifetimes, capture how ground-state polarisation is activated under excitation and translated into enhanced charge availability and utilisation.

#### Interfacial Polarisation Descriptors

While ground-state and excited-state descriptors define how polarisation is encoded in the framework and activated under excitation, its ultimate relevance to catalysis is realised at surfaces and interfaces, where charge transfer and chemical transformations occur. At this level, polarisation manifests as effective electric fields, vacuum-level offsets, and band bending that directly influence carrier drift, interfacial energetics, and reaction pathways. A central interfacial descriptor is the surface potential, which reflects the local electrostatic potential at the material surface relative to a reference [81, 82]. Variations in surface potential capture the projection of framework-level polarisation to reactive interfaces, providing insight into polarisation domains, field gradients, and interfacial electrostatic heterogeneity. Closely related is the work function (*Φ*), whose shifts indicate polarisation-induced modulation of the vacuum-level and energy-level alignment at COF surfaces. Together, surface potential and work-function descriptors quantify the strength and directionality of polarisation-induced interfacial electrostatics [83].

In liquid or electrochemical environments, the zeta potential (*ζ*) serves as an auxiliary descriptor of surface charge and electrostatic affinity, particularly for ionically modified or solvated COFs [84, 85]. *ζ* reflects the effective electrostatic environment experienced by ions near the surface and provides practical insight into how intrinsic polarisation persists under dielectric screening. However, *ζ* is strongly dependent on electrolyte composition, pH, and ionic strength, and quantitative comparisons require matched conditions.

It is important to emphasise that interfacial descriptors are complementary rather than interchangeable with ground—and excited-state metrics. A large intrinsic dipole does not necessarily lead to efficient exciton dissociation, just as reduced exciton binding energy alone does not guarantee favourable interfacial energetics or catalytic selectivity. Meaningful links between polarisation and function therefore emerge only when electronic, excitonic, and interfacial descriptors are considered together. This hierarchical descriptor framework provides the quantitative foundation for the polarisation-probing methodologies and validation strategies discussed in the following section.

### Probing Polarisation in COFs

Based on the hierarchical descriptor framework outlined above, reliable assessment of polarisation in COFs requires methodological validation across multiple length and time scales. Because polarisation in COFs is typically weak, spatially distributed, and predominantly electronic in origin, it cannot be inferred from a single structural or macroscopic signature. Instead, its presence and functional consequences must be established through the combined use of theoretical simulations and advanced experimental probes that connect intrinsic electrostatic descriptors with charge dynamics and interfacial behaviour under relevant conditions. In contrast to conventional ferroelectrics, where polarisation is often inferred directly from crystal symmetry or macroscopic hysteresis, polarisation in COFs must be validated through multiscale, multimodal characterisation. Accordingly, this section summarises the computational and experimental toolkits most widely employed to probe, cross-validate, and correlate polarisation descriptors with electronic structure, carrier dynamics, and interfacial energetics in COFs.

#### Theoretical Calculations

First-principles calculations, primarily density functional theory (DFT), provide the core quantitative basis for identifying polarisation and built-in electric fields in COFs [86, 87]. In most studies, polarisation is most frequently diagnosed using real-space descriptors rather than macroscopic ferroelectric formalisms. Because polarisation in COFs is typically weak, spatially distributed, and dominated by electronic rather than ionic displacement, it cannot be reliably described using macroscopic ferroelectric formalisms. Instead, polarisation is evaluated using a set of real-space and electronic-structure quantities directly accessible from DFT calculations and well suited to periodic, *π*-conjugated frameworks. At the most direct level, DFT provides access to the ground-state charge density and the corresponding ESP. By analysing the distribution of ESP and its spatial gradient within a periodic COFs layer, one can explicitly visualise internal potential contrasts induced by polar linkages, push–pull *π*-skeletons, or ionic motifs. When polarisation is predominantly in-plane, the in-plane potential drop (Δ*V*) extracted from ESP profiles is commonly used as a quantitative measure of the internal electrostatic driving force. This approach enables direct comparison across linkage conversions (e.g., imine to amide) or different polar motifs, without invoking macroscopic polarisation definitions.

From the same DFT charge density, dipole moments (or dipole density) can be calculated for the repeating unit via wavefunction post-processing [88]. It provides an intuitive measure of overall charge separation and captures the net polarisation effect within a periodic unit. It is particularly useful for isostructural series in which topology and crystallinity are conserved, such as in linkage conversion or substituent modification. Complementarily, charge-density difference maps, constructed by subtracting reference charge densities from the polarised framework, visualise the directionality and spatial extent of polarisation-induced charge redistribution. Charge-partition analyses, such as the Bader or Hirshfeld methods, further decompose the DFT charge density into atom- or bond-resolved contributions, enabling quantitative identification of charge accumulation/depletion (e.g., charge enrichment on imine N and depletion on adjacent C–H), thereby linking specific linkages or substituents to the emergent dipole. Beyond total charge density, polarisation effects are frequently examined through band-edge projections and decomposed charge densities (typically at the hybrid-functional level, as needed). These analyses reveal whether the CBM and VBM reside on spatially distinct subdomains, providing direct electronic-structure evidence for polarisation-programmed, type-II-like charge separation within single-component COFs.

To move beyond static electronic descriptors, polarisation-function correlations in COFs increasingly rely on dynamical simulations. Ab initio molecular dynamics (AIMD) calculations are widely used to assess thermal stability, structural robustness, and adsorption dynamics at finite temperatures [89, 90]. By capturing lattice fluctuations, framework flexibility, and adsorption dynamics under realistic conditions, AIMD provides essential validation that polarisation-induced structural motifs and electrostatic features persist beyond idealised static geometries. Non-adiabatic molecular dynamics (NAMD) calculations have also been used in several representative studies to estimate carrier recombination lifetimes and quantify how enhanced built-in fields suppress electron–hole overlap and prolong charge survival [91, 92]. Although computationally demanding, such simulations offer direct theoretical insight into how polarisation translates into enhanced carrier utilisation, complementing time-resolved spectroscopic measurements.

In addition, to connect polarisation to catalytic feasibility, DFT-based adsorption energetics and free-energy analyses are used to identify active sites and evaluate reaction thermodynamics, thereby bridging internal electrostatics with interfacial reaction coordinates [93]. In these calculations, adsorption energies are first evaluated by comparing the total energies of adsorbate-bound and clean surfaces, allowing identification of polarisation-stabilised active sites and preferred adsorption configurations. Changes in adsorption strength induced by built-in electric fields provide a direct measure of how polarisation modifies surface binding energetics. For multi-step electrochemical reactions such as hydrogen evolution reactions (HER) [94], or oxygen evolution reactions (OERs) [95], reaction free-energy diagrams are constructed by calculating the Gibbs free-energy change (Δ*G*) for each elementary step. These Δ*G* values are obtained from DFT total energies with standard zero-point energy and entropic corrections, often within the computational hydrogen electrode framework. The step with the largest uphill free-energy change is identified as the potential-determining (rate-limiting) step, from which the theoretical overpotential is derived. By comparing free-energy profiles across polarised and non-polarised COFs, these analyses reveal how built-in electric fields selectively stabilise key intermediates, lower reaction barriers, and shift rate-limiting steps, thereby translating polarisation effects into quantitative catalytic performance metrics.

#### Spectroscopic and Electrical Measurements

Experimental verification of polarisation effects in COFs typically relies on spectroscopic and electrical measurements that interrogate exciton dissociation, carrier separation/transport, and recombination pathways under working-relevant conditions. Unlike classical ferroelectrics, where spontaneous polarisation is often verified through macroscopic hysteresis measurements, polarisation in COFs is typically weak, electronically dominated, and spatially distributed. Consequently, its experimental confirmation relies on correlating surface-potential signatures with carrier dynamics and interfacial kinetic responses under working-relevant conditions. PL-based spectroscopies provide a primary window into polarisation-regulated recombination dynamics. Steady-state PL quenching is frequently observed in polarised frameworks, reflecting reduced radiative recombination due to field-assisted charge separation or more efficient carrier extraction to interfaces or cocatalysts. Time-resolved photoluminescence (TRPL) provides further kinetic fingerprints of polarisation-regulated recombination, allowing carrier lifetimes to be extracted by fitting the decay kinetics [96]. Depending on the dominant loss channel, polarisation engineering may manifest as prolonged, long-lived components (suppressed non-radiative recombination) or shortened emissive lifetimes due to faster interfacial extraction/charge transfer; therefore, TRPL should be interpreted together with transport and interfacial characterisations, such as photocurrent and electrochemical impedance spectroscopy (EIS) [97].

To directly capture ultrafast charge-transfer processes and long-lived charge-separated states, transient absorption spectroscopy (TAS) is increasingly used [98]. Operating in the picosecond-to-nanosecond regime, TAS tracks excited-state absorption and bleach recovery, providing real-time insight into how built-in electric fields accelerate charge separation and suppress it. When analysed alongside PL and TRPL, TAS enables a kinetic picture that links polarisation to carrier survival across multiple time scales. A distinctive excitonic bottleneck in conjugated COFs is the large *E*_*b*_, which limits the generation of free carriers. In several representative systems, temperature-dependent PL is employed to extract *E*_*b*_, providing an experimentally anchored descriptor for polarisation-enhanced exciton dissociation [99]. When combined with TAS/TRPL, the *E*_*b*_ trend offers a coherent picture linking polarisation to the transition from tightly bound excitons towards more weakly bound or spatially separated electron–hole pairs. Electrochemical and electrical characterisations provide complementary evidence for polarisation-assisted transport and interfacial kinetics. Transient photocurrent responses and photoelectrochemical measurements directly probe field-enabled carrier drift and extraction efficiency under illumination [100]. EIS is widely used to quantify interfacial charge-transfer resistance (*R*_ct_), with a smaller semicircle radius often correlating with stronger internal fields and faster charge delivery to reactive interfaces. Mott–Schottky analysis, when applicable, provides insight into flat-band potential shifts and changes in carrier density associated with polarisation-induced band bending or ionic/dipolar modification.

It is noteworthy that spectroscopic and electrical measurements primarily capture the consequences of polarisation rather than the electric field itself. Therefore, robust validation requires cross-correlation with surface-potential mapping and theoretical electrostatic descriptors, ensuring that observed carrier-dynamics improvements are indeed attributable to built-in electrostatic modulation rather than alternative structural or compositional effects.

#### Surface Potential and Local Field Mapping

Polarisation in COFs ultimately manifests as built-in electric fields and interfacial electrostatic force. Surface-potential and work-function measurements are among the most direct experimental tools for visualising polarisation-induced fields. Kelvin probe force microscopy (KPFM), including amplitude-modulated KPFM (AM-KPFM), measures the local contact potential difference between a conductive probe and the sample surface, thereby mapping spatial variations in surface potential with nanometer-scale resolution [81]. In polarised COFs, KPFM enables nanoscale mapping of contact potential difference and surface-potential contrast, thereby providing spatially resolved evidence for dipole alignment, ionic polarisation, or stacking- or topology-induced asymmetry. Its key advantage lies in spatial resolution, enabling identification of polarisation domains and heterogeneity within a single framework. However, KPFM is sensitive to surface roughness, conductivity, and environmental conditions and typically probes near-surface electrostatics rather than bulk polarisation; careful sample preparation and comparative analysis across isostructural systems are therefore essential. Complementarily, ultraviolet photoelectron spectroscopy (UPS) can quantify polarisation- or ion-pair-induced work-function shifts and vacuum-level offsets [101]. Polarisation-induced dipole layers or ion-pair motifs at the surface lead to measurable shifts in *Φ*, reflecting changes in interfacial ESP and energy-level alignment. UPS is particularly informative for post-synthetically modified or ionically functionalised COFs where dipole layers and local electrostatic stabilisation of frontier orbitals are invoked. Polarisation-induced dipole layers or ion-pair motifs at the surface lead to measurable shifts in *Φ*, reflecting changes in interfacial ESP and energy-level alignment.

In aqueous or electrolyte environments, *ζ*-potential measurements offer a convenient descriptor of surface charge and electrostatic affinity that often co-varies with ionic polarisation and adsorption behaviour [102]. *ζ*-potential reflects the effective surface charge at the slipping plane and often correlates with ionic polarisation, adsorption affinity, and electrostatic interactions with charged intermediates [103]. While the *ζ*-potential does not directly quantify internal electric fields, it provides a convenient, experimentally accessible descriptor of polarisation-modified surface electrostatics under working-relevant conditions. Its interpretation, however, is strongly dependent on electrolyte composition, pH, and ionic strength, and quantitative comparisons require matched conditions.

In polarised systems designed for oxygen reduction or pollutant degradation, mechanistic confirmation of polarisation-directed reaction pathways and selectivity is frequently strengthened by in situ/operando spectroscopies and electrochemical selectivity probes, such as electron paramagnetic resonance (EPR) for reactive oxygen species identification, scavenger tests to differentiate dominant radicals/intermediates, rotating ring-disc electrode (RRDE) analysis to quantify electron-transfer number and product selectivity (e.g., 2e⁻ vs. 4e⁻ ORR), and in situ diffuse reflectance infrared spectroscopy (DRIFTS) to track surface-bound intermediates [104–106]. Although these techniques do not map the electric field, they provide functional validation that polarisation-modified electrostatics translate into altered adsorption energetics, intermediates, and selectivity under catalytic conditions.

In some cases, piezoresponse force microscopy (PFM) and surface photovoltage (SPV) measurements have been applied to probe dipolar responses or photoinduced potential changes in COFs [107, 108]. SPV measurements probe changes in surface potential upon illumination, reflecting photoinduced charge separation, redistribution, and band bending at surfaces or interfaces. In polarised COFs, SPV can reveal how built-in electric fields facilitate directional charge separation upon light excitation, resulting in measurable photovoltage signals. PFM detects the electromechanical response of a material by monitoring surface deformation under an applied alternating electric field delivered through a conductive atomic force microscopy tip. In inorganic ferroelectrics, this response is directly linked to switchable spontaneous polarisation. In COFs, however, any observed PFM signal more plausibly originates from field-induced dipolar alignment, ionic displacement, electrostatic forces, or flexoelectric effects, rather than from true ferroelectric switching. As a result, PFM measurements in COFs are best interpreted as qualitative indicators of dipolar or polarisable behaviour. Given the weak polarisation and soft lattice nature of most COFs, such measurements should generally be framed as supportive/auxiliary evidence of dipolar responses or field effects, rather than definitive proof of ferroelectric switching, unless a clear hysteresis/phase response with rigorous artefact controls is provided.

In summary, by jointly integrating theoretical calculations with spectroscopic, electrical, and surface-sensitive measurements, polarisation in COFs can be understood as a quantitatively definable and experimentally traceable electrostatic phenomenon rather than a qualitative structural descriptor. Theory establishes the electronic origins and relevant polarisation parameters, while complementary probes connect these descriptors to charge separation, transport, and interfacial energetics under working conditions. Crucially, surface-potential and local-field-sensitive measurements anchor polarisation effects at catalytically relevant interfaces, revealing how built-in electrostatic fields reshape band bending, reaction energetics, and ultimately turnover behaviour.

## Strategies for Polarisation Engineering

Building on the above discussion of the generation mechanism and methods to identify polarisation in COFs, this section examines representative strategies employed in catalysis to illustrate how polarisation in COFs is achieved. We classify these widely applied strategies based on the structural origin of polarisation, spanning bond-level, lattice-level, and post-synthetic modifications. For each category, the dominant polarisation features, such as electrostatic potential gradients, dipole density, and surface-potential contrast, are linked to their influence on exciton and charge-carrier behaviour as well as interfacial catalytic processes. Table 1 summarises the polarisation regulation strategies commonly used in COFs, the physical or electronic descriptors used to characterise them, and the catalytic limitations they are typically applied to overcome.

**Table 1:** Summary of design rules for inducing polarisation in COFs and the associated electronic descriptors

Regulation Level	Polarisation strategy	Structural origin of polarisation	Dominant polarisation descriptor affected	Descriptor most strongly tuned	Catalytic limitations primarily alleviated	References
Bond Level	Linkage polarity engineering	Intrinsic bond dipoles aligned through *π*-conjugation (e.g., C=N, C=O, zwitterionic resonance)	In-plane electrostatic potential gradient	Exciton binding energy (*E*_*b*_)	Strong excitonic confinement in low-dielectric COFs	[63, 76, 109–111]
	Atomic/heteroatom substitution	Local electronegativity contrast without topology change	Local dipole magnitude	*E*_*b*_ and carrier delocalisation	Limited exciton dissociation despite extended conjugation	[112]
	Linkage orientation inversion	Reversal of polar bond vector at constant crystallinity	Dipole vector direction	*E*_*b*_ versus transport balance	Ambiguous causality between structure and polarity	[51, 113, 114]
	Substituent polarity grading	Superposition of local dipoles on a fixed backbone	Effective internal field (non-monotonic)	Adsorption free energy versus *E*_*b*_	Trade-off between charge separation and intermediate binding	[113, 115]
Framework Level	D–A *π*-skeletons	Push–pull charge deformation across repeating D–A motifs	Net dipole density and internal field strength	Real-space band-edge separation	Poor directionality of charge transport	[55, 73, 116–118]
	Defect-enabled polarisation	Local D–A microdomains embedded in periodic lattice	Spatially heterogeneous field enhancement	Local charge separation rate	Insufficient local charge density at reaction sites	[119]
	Multipolar symmetry control	Symmetry-enforced cancellation of net dipole with strong ICT	Exciton delocalisation length	Reduced *E*_*b*_ without net dipole	Exciton trapping despite large molecular dipoles	[120, 121]
	Non-centrosymmetric topology	Lattice-scale inversion symmetry breaking	Macroscopic polarisation field	Directional carrier drift	Lack of intrinsic transport anisotropy	[122, 123]
Interface Level	Polar side-chain functionalisation	Dipolar side groups coupled with pore microenvironment	Field expression under dielectric screening	Interfacial charge-transfer kinetics	Inefficient charge utilisation in aqueous media	[124]
	Post-synthetic ionic modification	Fixed ion pairs forming internal dipole layers	Built-in electric field magnitude	Band-edge alignment and *E*_*b*_	Weak internal field in neutral frameworks	[1]
	Post-synthetic dipolar junctions	Periodic molecular dipoles grafted onto backbone	Local potential discontinuity	Charge extraction efficiency	Limited separation in otherwise nonpolar lattices	[125]
	Functional group conversion	Polar defect generation with ion-enrichment	Extended electrostatic field range	Ion-charge coupling	Decoupled adsorption and electron transfer	[126]
	Oxidation-induced ionic motifs	Zwitterionic/ionic units altering local electrostatics	Intermediate stabilisation field	Δ*G*(*intermediate)	Poor pathway selectivity in multi-electron reactions	[67]

### Linkage Polarity Tuning

The polarity of covalent linkages provides one of the most direct chemical approaches for programming polarisation in COFs, because linkages simultaneously govern three interrelated aspects: (i) the magnitude and orientation of bond dipoles, (ii) the continuity and directionality of π-conjugation, and (iii) the microscopic charge distribution that ultimately manifests as an intrinsic ESP gradient across the lattice. Accordingly, increasing electronegativity contrast within linkages by incorporating polar motifs, such as imine (C=N) [63, 64, 113, 114], hydrazone (C=N–NH) [65], amide (C(O)NH) [109], thiazole (C–N–S) [127], and boramidic-acid (B–N) [110] can strengthen intrinsic polarisation, leading to built-in electric fields that lower E_b_ and promote exciton dissociation and directional charge separation. Notably, linkage polarity should not be regarded as a purely local structural feature; once bond dipoles are periodically embedded within a crystalline framework, their collective alignment can be amplified into a framework-scale potential landscape that, in turn, governs recombination kinetics, interfacial charge transfer, and even adsorption/activation at catalytic sites.

**Fig. 4:**
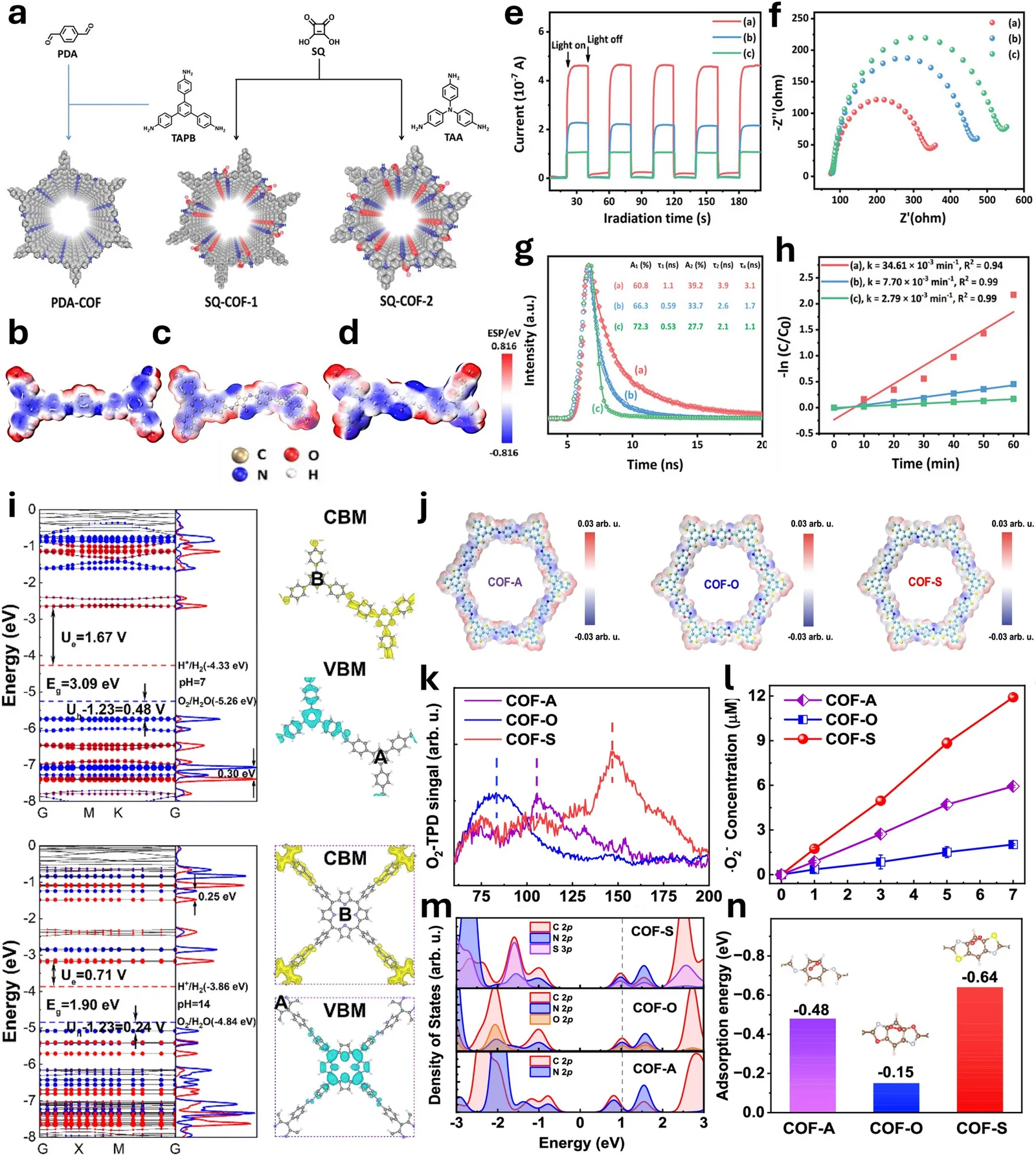
**a** Schematic synthesis of SQ-COF-1, SQ-COF-2, and PDA-COF. **b-d** High magnification ESP maps in the ground state. Comparison of **e** photocurrent responses, **f** EIS Nyquist plots, **g** TRPL, and **h** visible-light photocatalytic degradation of SM2 for the three COFs. Reproduced with permission [76]. Copyright 2022, Wiley. **i** HSE06 band structures of Im-TPB and Im-TPP projected onto two symmetry-related subdomains (Core A and Core B), illustrating a type-II-like band alignment. The corresponding CBM and VBM charge-density distributions are shown on the right. Reproduced with permission [63]. Copyright 2024, RSC. **j** ESP maps of COF-A, COF-O, and COF-S, showing linkage-dependent charge polarisation. **k** O_2_ temperature-programmed desorption profiles reflecting oxygen adsorption strength. **l** Relative ·O_2_^−^ generation under visible-light irradiation. **m** PDOS highlighting electronic modulation induced by different linkages. **n** Calculated O_2_ adsorption energies. Reproduced with permission [111]. Copyright 2024, Springer Nature

Ben and co-workers reported a prototype example of linkage-induced polarisation in a squaraine-linked COF (SQ-COF) [76]. SQ-COF-1 was synthesised by condensing squaric acid with 1,3,5-tris(4-aminophenyl)benzene (TAPB) to form a photoactive squaraine linkage, as shown in Fig. 4a. To isolate the specific contribution of linkage polarity, an isostructural series of COFs was also prepared by systematically varying the linkage chemistry while preserving the overall framework topology, including SQ-COF-2 with a tertiary amine-modified linkage and a non-zwitterionic imine-linked analogue (PDA-COF). Ground-state ESP maps provide a direct visualisation of how the SQ linkage establishes a polarity gradient: in SQ-COF-1 (Fig. 4b), a pronounced potential gradient is observed, with positive electrostatic potential localised on the TAPB segment, whereas negative electrostatic potential is concentrated on the oxygen atoms of the SQ moiety. By contrast, introducing a tertiary amine in SQ-COF-2 (Fig. 4c) partially neutralises the positive potential, and PDA-COF exhibits a comparatively homogeneous ESP distribution (Fig. 4d). Consistent with this ESP trend, SQ-COF-1 displays the largest molecular dipole moment (2.75 Debye) and the highest surface potential. Notably, the zwitterionic linkage also increases the interlayer spacing due to electrostatic repulsion. This phenomenon demonstrates that linkage polarity not only defines the internal electrostatic landscape but also can couple to framework stacking and packing, thereby modulating the internal field. Functionally, this linkage polarity enhanced polarisation manifests as more efficient carrier separation and significantly reduced charge recombination, as evidenced by a larger transient photocurrent (Fig. 4e), a lower interfacial *R*_ct_ (Fig. 4f), and an extended TRPL lifetime (Fig. 4g). As a result, SQ-COF-1 achieves complete degradation of SM2 (25 ppm) within 60 min, with reaction rates ~ 4.5 × and ~ 12.4 × higher than those of SQ-COF-2 and PDA-COF, respectively (Fig. 4h), along with high mineralisation efficiency and excellent stability.

Beyond specific experimental case studies, theoretical studies have demonstrated that linkage polarity can act as an intrinsic design parameter for programming internal fields and band alignment in COFs. Using first-principles calculations, Fan et al. demonstrated that the asymmetric charge distribution of the imine bond (–CH=N–) can induce an in-plane dipole, splitting the electronic environments of two homogeneous cores within a 2D monolayer and producing a type-II-like alignment [63]. As illustrated by the projected band structures and real-space band-edge charge densities (Fig. 4i), the CBM and VBM become spatially localised on distinct subdomains, despite the absence of explicit donor–acceptor pairing at the building-block level. This behaviour originates from a linkage-originated dipole field, as further supported by Bader charge analysis, and as a direct consequence, a pronounced suppression of electron–hole recombination is observed, as evidenced by substantially prolonged carrier lifetimes relative to nonpolar azo-linked analogues. Subsequently, the same group demonstrated that increasing linkage polarity provides a direct route to amplifying charge separation [109]. Conversion of the imine bond to a more polar amide linkage (–C(O)N–) strengthens the in-plane dipole and deepens the potential differentiation between subdomains, with the calculated in-plane potential drop increasing from 0.32 to 0.72 eV. This enhanced potential landscape translates into markedly improved carrier dynamics, including longer recombination lifetimes (14.86 vs. 4.31 ns) and a 3.5-fold increase in charge-separation efficiency, while simultaneously repositioning the band edges to satisfy the thermodynamic requirements for spontaneous overall water splitting. More recently, Fan’s group extended this strategy to a boramidic-acid linkage in a 2D COF (COF-BHT), where a highly polarised bond splits band levels across structural subdomains to form a dual-type-II alignment, enabling ultrafast inter-domain charge transfer on the femtosecond-picosecond timescale while maintaining nanosecond-scale recombination lifetimes [110]. Thermodynamic analyses place the conduction and valence edges suitably straddling H^+^/H_2_ and O_2_/H_2_O, implying overall water splitting in pure water without cocatalysts, with a modest theoretical STH efficiency of 5.10%. In summary, these theoretical studies elevate linkage polarity tuning from a qualitative guideline to a quantitative electronic design strategy, in which the magnitude of in-plane potential differentiation and the spatial localisation of band-edge states serve as predictive descriptors for charge-separation efficiency and recombination kinetics. Importantly, the predictive linkage-dipole framework has been experimentally validated through orientation-controlled imine engineering [51]. Constitutional isomerisation of C=N bonds within an isostructural D–A COF platform was shown to modulate the cooperative alignment between imine dipoles and D–A asymmetry, thereby amplifying the in-plane built-in electric field without altering backbone topology. The optimised configuration exhibited a ~ 50% increase in framework dipole moment, accompanied by a marked reduction in exciton binding energy and a sevenfold extension of carrier lifetime, confirming that linkage-level dipole orientation is key for programming framework-scale electrostatic landscapes and charge-carrier kinetics.

While linkage polarity provides an effective way to strengthen bond dipoles, maintaining such polarised electronic structures under realistic aqueous conditions remains a non-trivial challenge. One practical approach is to aromatise dynamic imine (C=N) linkages into rigid heteroaromatic motifs, thereby preserving polarised electronic structures while simultaneously improving framework stability. In a recent study, Tong’s team reported a representative example in which an imine-linked COF (COF-A) was transformed into oxazole- and thiazole-linked analogues (COF-O and COF-S) through a one-pot topochemical cyclisation that preserves long-range crystallinity while redistributing the charge density around the linkage [111]. Notably, the linkage rigidification directly addresses aqueous robustness, in which the aromatised oxazole/thiazole linkages suppress hydrolysis-prone dynamics of imines and endow the frameworks with markedly enhanced chemical stability, as evidenced by the retained crystallinity/structural signatures after exposure to harsh acidic/alkaline conditions and by the preserved catalytic activity upon consecutive reuse under aqueous operation. Such structural integrity provides the prerequisite for sustaining the electrostatic potential contrast and the associated built-in field effects in water, rather than merely strengthening the intrinsic dipoles. ESP maps in Fig. 4j reveal a progressive enhancement of local charge polarisation from COF-A to COF-O and further to COF-S, with the thiazole linkage generating the most pronounced polarity contrast along the framework backbone. Consistent with the enhanced linkage polarisation, COF-S exhibits a substantially reduced E_b_ and the most favourable charge-separation and transport characteristics, reflecting more efficient dissociation of photogenerated carriers.

In addition to influencing bulk charge dynamics, enhanced linkage polarity also exerts a decisive influence at the catalyst–reactant interface by reshaping the local electronic environment for O_2_ adsorption and activation. Oxygen temperature-programmed desorption experiments demonstrate markedly stronger O_2_ adsorption on COF-S (Fig. 4k), which correlates with a significantly higher yield of superoxide radicals (·O_2_^−^) under visible-light irradiation (Fig. 4l). Projected density of states (PDOS) analysis suggests that the thiazole linkage optimises the local electronic structure of adjacent carbon sites (Fig. 4m), leading to a more favourable O_2_ adsorption energy than that of the imine and oxazole counterparts (Fig. 4n). This polarised thiazole microenvironment thus promotes efficient O_2_ activation and subsequent release of reactive ·O_2_^−^ species, with photogenerated holes providing an auxiliary oxidative pathway during pollutant degradation. Functionally, these synergistic effects enable COF-S to achieve near-quantitative degradation of paracetamol within minutes under visible light and to outperform both imine—and oxazole-linked analogues across a range of emerging contaminants. Notably, the rigid heteroaromatic linkage also confers superior chemical stability, allowing COF-S to retain high activity across broad pH windows and complex water matrices, as well as after immobilisation in continuous-flow and enlarged reactor configurations under light irradiation.

### Donor–Acceptor Framework Design

Introducing donor (D) and acceptor (A) motifs into a periodic lattice offers a framework-level route to impose long-range charge asymmetry in COFs. Whereas linkage polarity tuning (Sect. 3.1) primarily programs bond-scale dipoles, D–A design operates at the motif and lattice scales to construct an intraframework push–pull potential landscape along the *π*-skeleton, thereby offsetting frontier orbital energies and generating built-in electric fields that bias directional carrier migration. In practice, the goal of D–A engineering is not merely the coexistence of donors and acceptors, but the quantitative control of the internal field strength and real-space charge separation. When properly optimised, D–A COFs can approach type-II-like charge separation within a single crystalline lattice, where the spatial localisation of CBM and VBM mimics heterojunction behaviour without forming a physical interface.

**Fig. 5:**
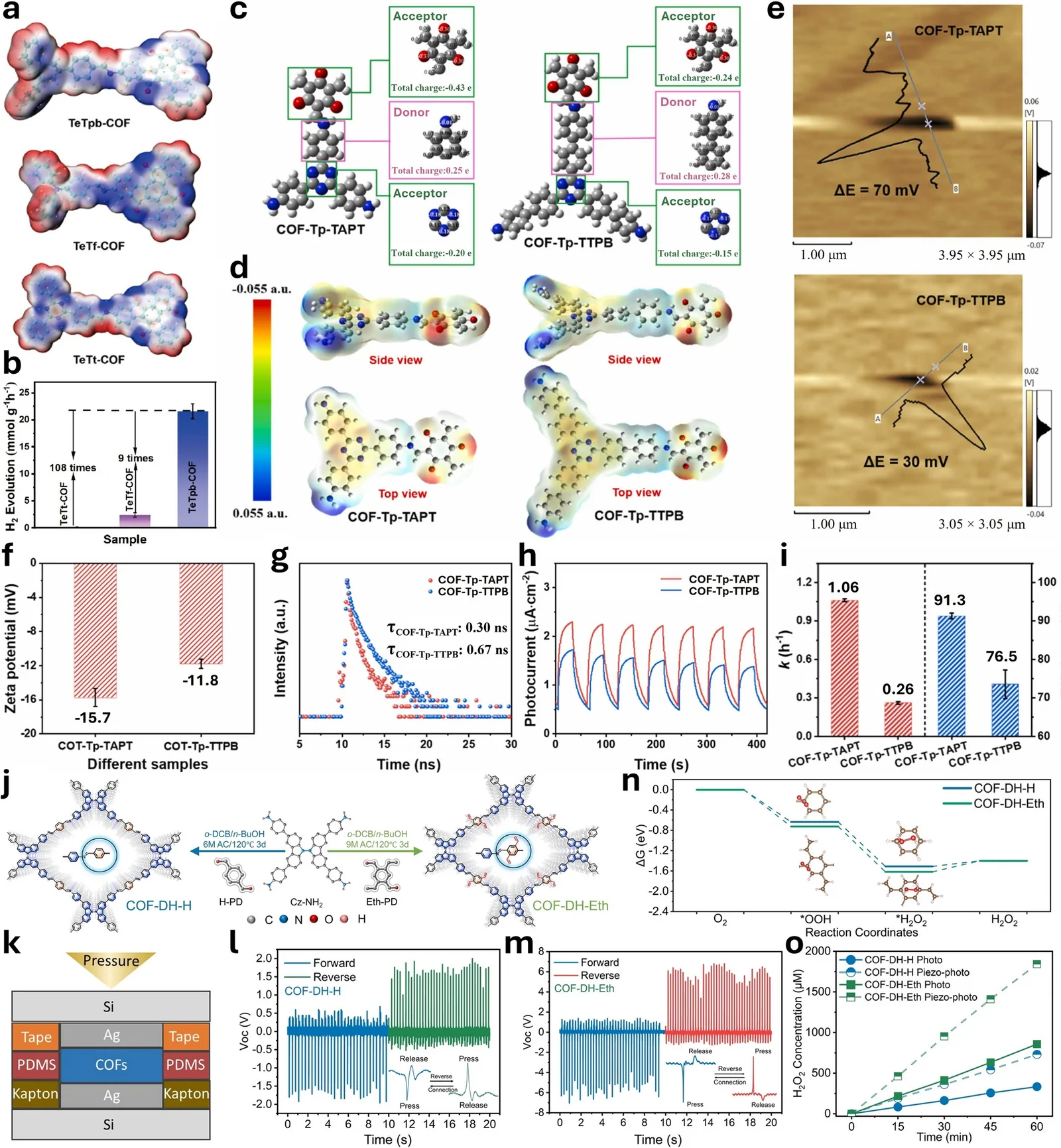
**a** Electrostatic potential distributions of TeTpb-COF, TeTf-COF, and TeTt-COF, where blue and red regions denote electron accumulation and depletion, respectively. **b** Comparison of photocatalytic hydrogen evolution rates of Te-COFs under identical conditions. Reproduced with permission [73] Copyright 2024, Springer Nature. **c** Hirshfeld charge analysis of COF-Tp-TAPT and COF-Tp-TTPB. **d** Calculated surface electrostatic potential distributions of representative framework fragments in the two COFs. **e** KPFM surface-potential maps of COF-Tp-TAPT and COF-Tp-TTPB; insets show the corresponding surface-potential line profiles. **f** Zeta-potential measurements of COF-Tp-TAPT and COF-Tp-TTPB. **g** TRPL decay profiles. **h** Transient photocurrent responses recorded under intermittent light irradiation. **i** Photocatalytic ciprofloxacin degradation performance of the two COFs. Reproduced with permission [116] Copyright 2025, Elsevier. **j** Synthetic schemes illustrating the construction of COF-DH-H and COF-DH-Eth. **k** Conceptual illustration of the piezoelectric nanogenerator configuration; corresponding forward and reverse open-circuit voltage outputs recorded for **l** COF-DH-H and **m** COF-DH-Eth under mechanical excitation (insets show locally magnified voltage profiles). **n** Calculated Gibbs free-energy profiles for the two-electron oxygen reduction pathway to H_2_O_2_ on the electron-enriched benzene site (site C) of COF-DH-H and COF-DH-Eth. **o** Comparison of H_2_O_2_ generation performance under photocatalytic and coupled piezo-photocatalytic conditions for COF-DH-H and COF-DH-Eth. Reproduced with permission [55]. Copyright 2025, Wiley

A representative example of framework-level field-strength engineering via D–A modulation was reported by Hao et al., who constructed a series of imine-linked D–A COFs (TeTpb-, TeTf-, and TeTt-COF) by integrating a strongly electron-withdrawing truxenone (Te, three C=O units) acceptor core with systematically varied aryl donor motifs [73]. The carbonyl-rich truxenone unit acts as an electron-withdrawing acceptor, whereas the aryl donors push charge back, establishing a conjugation-guided push–pull field. This intraframework field is directly visualised by Multiwfn-based electrostatic potential and charge analyses (Fig. 5a). Importantly, variation of donor motifs enables systematic modulation of framework polarity, with the dipole moment increasing to 8.28 Debye for TeTpb-COF, compared with 2.54 Debye and 2.28 Debye for TeTf- and TeTt-COF, respectively. Consistent with this enhanced polarisation, TeTpb-COF exhibits the longest-lived excited states and the lowest charge-transfer interfacial resistance, reaching a built-in electric field of 0.268 mV m^−1^ (vs. 0.061/0.058 mV m^−1^ for TeTf/TeTt). Correspondingly, the photocatalytic hydrogen evolution rate scales monotonically with the dipole strength across the series (Fig. 5b), reaching 21.6 mmol g^−1^ h^−1^ for TeTpb-COF, which is over two orders of magnitude higher than that of TeTt-COF. In this system, donor selection serves as a quantitative knob to tune the polarisation field, which manifests in suppressed recombination, accelerated interfacial charge transfer, and enhanced hydrogen evolution activity.

Building on donor-tuned D–A fields, Dong and co-workers further strengthened intraframework charge asymmetry by moving to an A_1_–D–A_2_ motif, in which quinone and triazine act as dual acceptors flanking an aryl donor segment in β-ketoenamine COFs (COF-Tp-TAPT and COF-Tp-TTPB) [116]. In this pull–push-pull configuration, polarisation is expected to be reinforced not by increasing donor strength, but by multiplying electron-withdrawing sinks that deepen the electrostatic potential drop along the conjugated pathway. DFT-derived Hirshfeld charge partitioning and electrostatic potential mapping (Fig. 5c, d) substantiate this mechanism, showing electron accumulation on the quinone/triazine domains and corresponding depletion on the phenyl/biphenyl donor. Quantitatively, this acceptor-multiplication strategy results in a higher framework polarity density, with the phenyl-donor COF-Tp-TAPT exhibiting a higher intramolecular polarity density (0.018 vs. 0.013 D Å^−2^) and a larger molecular polarity index (0.65 vs. 0.59 eV) than COF-Tp-TTPB. Importantly, this polarity enhancement is corroborated by framework-level probes: KPFM reveals a larger surface-potential contrast for COF-Tp-TAPT (ΔE ≈ 70 mV) relative to COF-Tp-TTPB (≈ 30 mV) (Fig. 5e), while the more negative zeta potential (− 15.7 vs. − 11.8 mV) indicates increased surface charge density (Fig. 5f). These strengthened fields translate into faster charge-separation/transfer, including suppressed emission, a shorter TRPL lifetime (0.30 ns vs 0.76 ns; Fig. 5g), and higher photocurrent response (Fig. 5h), alongside reduced interfacial transport resistance. As a result, COF-Tp-TAPT achieves 91.3% ciprofloxacin degradation with k = 1.06 h^−1^, outperforming COF-Tp-TTPB (76.5%, k = 0.26 h^−1^; Fig. 5i). Collectively, these results establish that dual acceptor domain engineering can effectively amplify the intraframework potential gradient, which can be quantitatively read out as larger surface-potential contrast, accelerated carrier kinetics, and improved photocatalytic turnover.

Apart from static D-A polarisation, recent studies have begun to explore D–A COFs as stimulus-responsive polarisation platforms, in which an externally applied polarised field cooperates with intrinsic charge-transfer asymmetry to bias carrier transport and reaction selectivity. For example, Liu’s team developed bicarbazole-based COFs featuring a D_1_–A–D_2_ charge-transfer motif (COF-DH-H and COF-DH-Eth; Fig. 5j) and demonstrated piezo-photocatalytic H_2_O_2_ synthesis by coupling visible-light excitation with ultrasonication [55]. Under ultrasound, a piezoelectric polarised field is generated and superimposed onto the intrinsic photoinduced driving force (Fig. 5k–m), resulting in more efficient carrier separation and faster interfacial electron transfer. Time-resolved PL and EIS measurements corroborate suppressed recombination and reduced charge-transfer resistance in the coupled mode. Beyond charge-transport enhancement, the externally reinforced polarisation field also reshapes the reaction thermodynamics at the catalyst-reactant interface. DFT charge-density difference and free-energy analyses reveal that the reinforced polarisation field reshapes the ORR energy landscape, facilitating *OOH formation while avoiding over-stabilisation of *O, thereby lowering the thermodynamic barrier towards the 2e⁻ pathway (Fig. 5n). As a result, COF–DH-Eth delivers an H_2_O_2_ formation rate of 9212 μmol g^−1^ h^−1^ under the coupled “piezo + light” mode, compared with 3840 μmol g^−1^ h^−1^ under light alone, while maintaining a two-electron ORR pathway (Fig. 5o). Such stimulus-reinforced polarisation illustrates that D–A COFs can transcend static charge-separation scaffolds and actively regulate reaction thermodynamics through field modulation. Importantly, the same “field-amplification” logic can also be realised intrinsically through structural programming of the D–A lattice, where charge localisation and excited-state stabilisation are reinforced without external stimuli. In this regard, Qiu and co-workers demonstrated that embedding carbonyl-enriched acceptor motifs into an imine-linked D–A COF (TroTfb-COF) intrinsically strengthens charge localisation and *π*–*π* stacking cooperativity, thereby extending light absorption into the red region (E_g_ ~ 1.91 eV) and enabling highly selective ^1^O_2_ generation under low-energy irradiation [118]. The spatially locked conformation and intensified interlayer coupling jointly stabilise charge-transfer excited states and suppress competing pathways, allowing efficient red-light-driven oxidation with markedly enhanced selectivity relative to the non-carbonyl analogue. Notably, intrinsic amplification of D–A polarisation is not limited to conformational locking, but can also be achieved via dynamic electronic reorganisation of the linkage environment. Zhang and co-workers recently showed that acceptor-strength tuning can modulate the keto-enol tautomeric equilibrium in TP-based D–A COFs, shifting the framework towards a keto-dominant (enaminone) configuration that enhances *π*-delocalisation continuity and promotes more efficient charge separation [117]. The optimised sulfone-containing COF exhibits prolonged exciton lifetimes (2.4 ns) and reduced charge-transfer resistance, translating into consistently improved ROS generation and superior performance across multiple aerobic oxidation transformations.

### Atomic and Substituent Modulation

Even when the primary linkage chemistry and backbone topology are fixed, polarisation in COFs can still be further tailored through local atomic and substituent modulation. In contrast to linkage polarity tuning and D–A framework design, which operate at the bond and lattice scales, this strategy serves as a local, modular control layer that perturbs electronic polarisation without compromising crystallinity or long-range order. In practice, such local modulation can be readily achieved through chemically accessible handles, including heteroatom substitution [76, 128, 129], controlled defect engineering [119, 130, 131], and side-chain functionalisation [132, 133]. Importantly, the effects of these local perturbations can be evaluated using a consistent set of polarisation and kinetic descriptors, allowing direct comparison across different COF platforms.

**Fig. 6:**
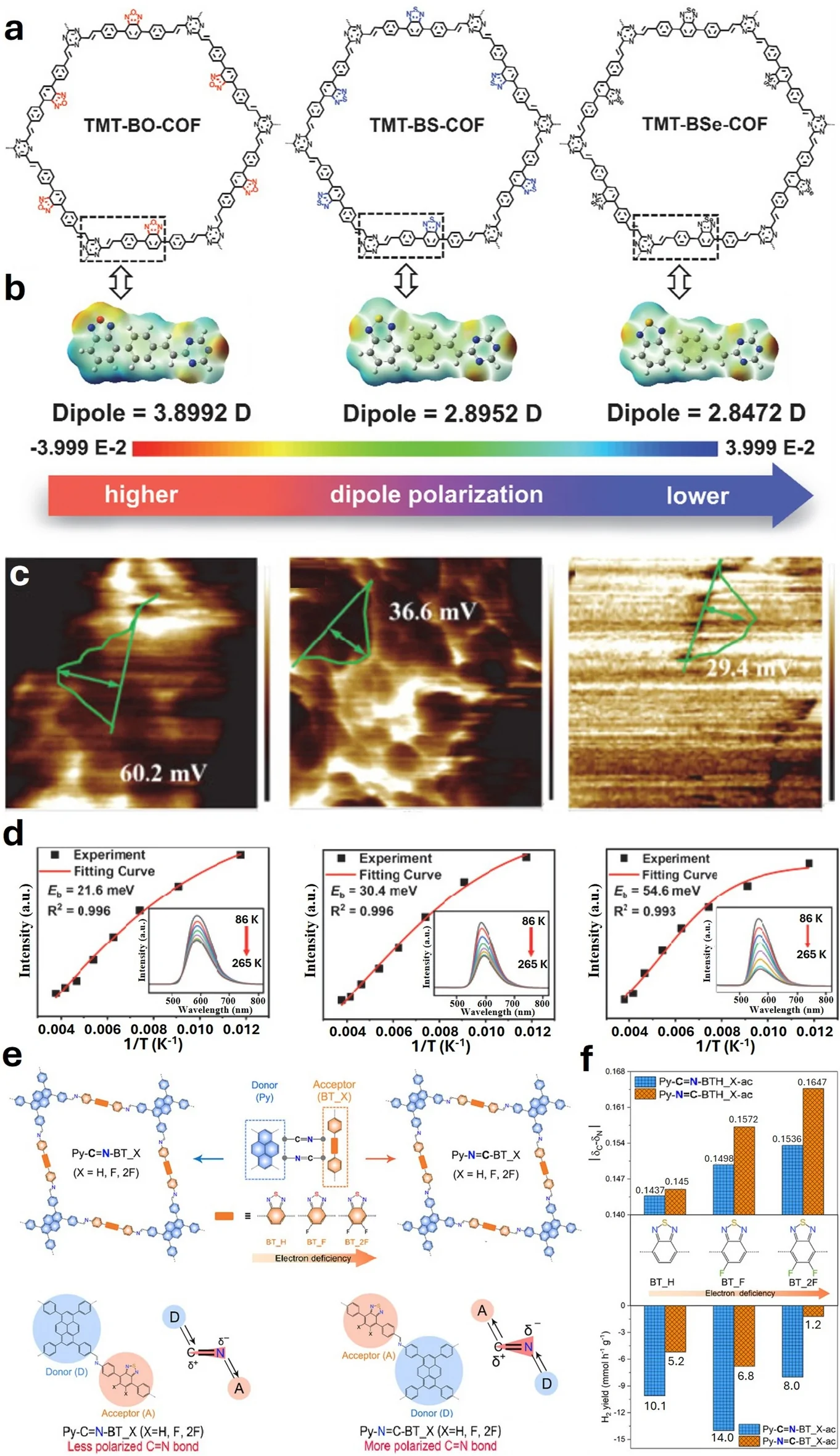
**a** Structural models of TMT-BO-COF, TMT-BS-COF, and TMT-BSe-COF. **b** ESP distributions and corresponding molecular dipole moments, illustrating graded dipole polarisation induced by O, S, and Se substitution. **c** KPFM surface-potential maps of the three COFs; green lines indicate the regions used for surface-potential profiling. **d** Temperature-dependent photoluminescence spectra (excitation at 400 nm) and corresponding Arrhenius analyses used to extract exciton binding energies (insets). Reproduced with permission [112]. Copyright 2024, Elsevier. **e** Chemical structures of Py-C=N-BT___X and Py-N=C-BT___X (X=H, F, 2F), illustrating imine-bond orientation-dependent modulation of C=N polarity; Py-C=N-BT___X with less polarised C=N bonds and Py-N=C-BT___X with more polarised C=N bonds are shown in the lower panels. **f** Correlation between photocatalytic hydrogen evolution activity and C=N bond polarity for all protonated D–A COFs. Reproduced with permission [113]. Copyright 2025, Wiley

Heteroatom substitution provides a particularly clean single-variable platform to correlate electronegativity-driven local dipoles with exciton physics and photocatalytic performance. Gao and co-workers constructed an isostructural series of vinylene-linked COFs by replacing the heteroatom in the benzodiazole unit with O, S, and Se, yielding TMT-BO-COF, TMT-BS-COF, and TMT-BSe-COF, respectively (Fig. 6a) [112]. The calculated molecular dipole moment decreases systematically from O to S and Se (3.9, 2.9, and 2.85 Debye, Fig. 6b), demonstrating graded polarisation modulation without altering the framework topology. This trend is experimentally reflected in the built-in electric field strength, as KPFM measurements reveal the largest surface-potential contrast for TMT-BO-COF (60.2 mV), consistent with more effective field-assisted carrier separation (Fig. 6c). Concomitantly, the *E*_*b*_ decreases markedly from 54.6 meV (Se) to 30.4 meV (S) and further to 21.6 meV (O) (Fig. 6d), indicating facilitated exciton dissociation. Multiwfn analysis supports this interpretation by showing progressively enhanced electron delocalisation in the LUMO (EDI: 8.19 > 8.11 > 8.09), which is conducive to charge separation and transport. These coupled electronic effects translate into a pronounced activity enhancement in photocatalytic H_2_ evolution, with TMT-BO-COF reaching 23.7 mmol g^−1^ h^−1^, surpassing TMT-BS-COF (9.45 mmol g^−1^ h^−1^) and TMT-BSe-COF (0.96 mmol g^−1^ h^−1^) under identical conditions. DFT calculations further indicate that triazine nitrogen sites act as key reduction centres and support a proton-coupled electron-transfer pathway, linking local dipole polarisation directly to intrinsic reduction kinetics rather than purely light-harvesting effects.

At the level of atomic and substituent modulation, polarisation effects in COFs no longer follow simple monotonic relationships, but instead expose intrinsic trade-offs among polarisation strength, exciton binding, and charge-transport pathways. Li et al. addressed this complexity by developing an isostructural D–A imine COF series in which the local C=N bond polarity is co-varied through a dual strategy: (1) constitutional isomers that control bond orientation (Py-C = N-BT vs. Py-N=C-BT) and (2) substituent modulation via fluorination of the acceptor unit (BT_H, BT_F, BT_2F). This design enables systematic tuning of the local dipole field while preserving the overall framework topology, as illustrated in Fig. 6e [113]. Interestingly, the less-polar Py-C=N-BT isomers (donor-to-carbon linkage) exhibit substantially lower E_b_ (40.95/49.66/52.58 meV for H/F/2F) than their more-polar Py-N=C-BT counterparts (65.60/71.98/78.10 meV). In contrast, increasing C=N polarity strengthens the internal electric field and charge-transfer characteristics, such that balancing these opposing effects leads to an optimal intermediate polarity, in which Py-C=N-BT_F-ac delivers the highest H_2_ evolution rate (14.0 mmol g^−1^ h^−1^), outperforming both the less-polar H analogue (10.1 mmol g^−1^ h^−1^) and the more-polar 2F variant (8.0 mmol g^−1^ h^−1^) (Fig. 6f). Such a polarisation–performance relationship demonstrates that atomic and substituent modulation do not aim for maximal polarity, but instead require matching local dipole strength to the dominant kinetic bottleneck.

**Fig. 7:**
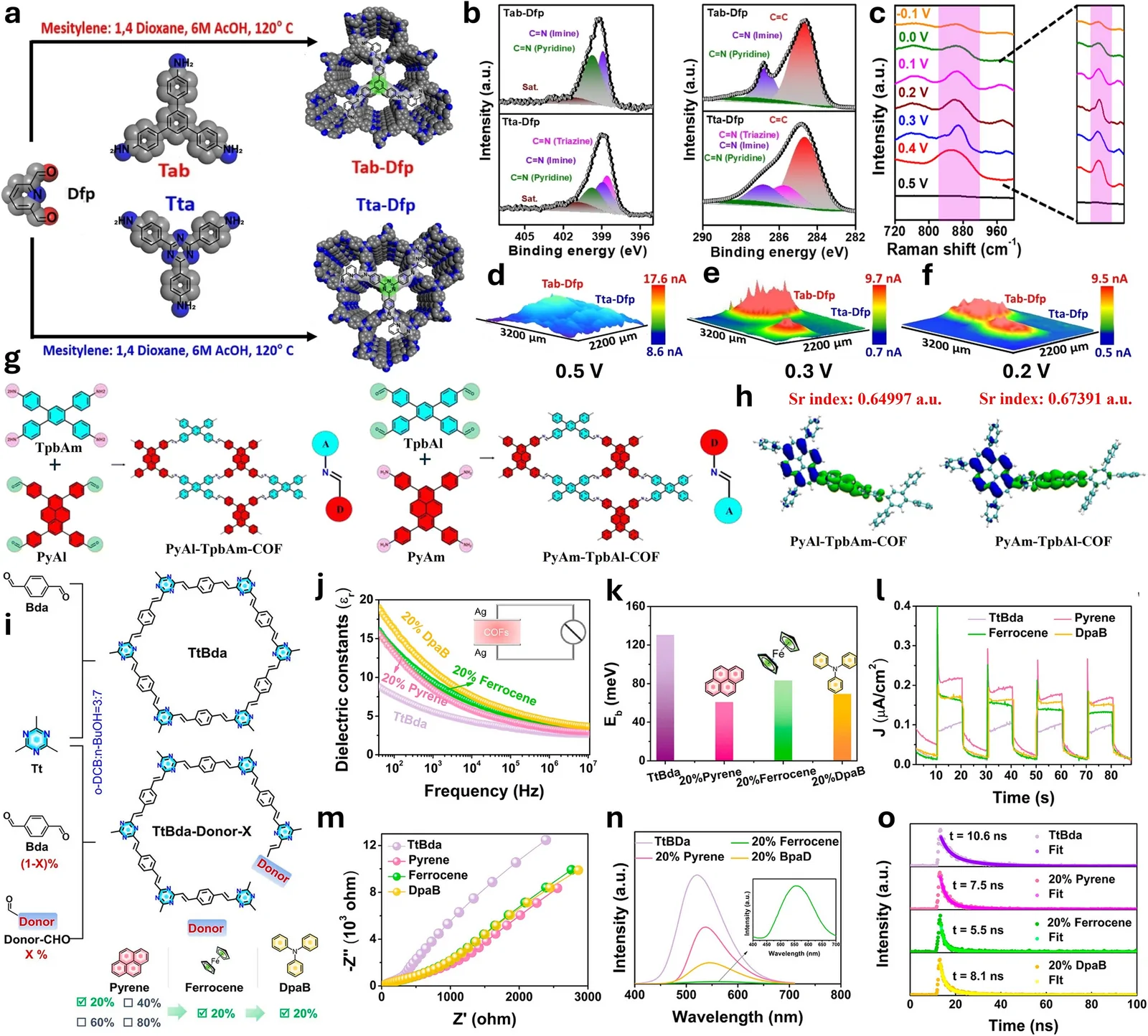
**a** Synthetic schemes of Tab-Dfp and Tta-Dfp. **b** Deconvoluted N 1s and C 1s XPS spectra. **c** In situ Raman spectra recorded during chronoamperometric measurements at applied potentials from 0.5 to − 0.1V. **d–f** 3D SECM images collected in 0.1M PBS at applied potentials of 0.5, 0.3, and 0.2V, respectively, with the tip polarised at 1.1V versus RHE. Reproduced with permission [134]. Copyright 2025, Wiley. **g** Synthesis routes of PyAl-TpbAm-COF and PyAm-TpbAl-COF. **h** DFT-calculated excited-state electron–hole distributions illustrating polarity modulation induced by imine orientation. Reproduced with permission [114] Copyright 2024, RSC. **i** Schematic illustration of donor-capped defect engineering in TtBda. **j** Frequency-dependent dielectric constants, **k** exciton binding energies, **l** photocurrent responses, **m** Nyquist plots, **n** steady-state PL spectra, and **o** TRPL decay spectra of pristine TtBda and donor-capped TtBda-X COFs. Reproduced with permission [119] Copyright 2023, ACS

Importantly, local polarity modulation in COFs can extend beyond accelerating charge dynamics to reshape adsorption configurations and reaction barriers, thus suppressing competing multi-electron pathways and improving selectivity. In the 2e⁻ oxygen reduction reaction (ORR), for example, the adsorption free energy of the *OOH intermediate (Δ*G*(*OOH)) is commonly used as a central descriptor for rationalising activity and selectivity trends between H_2_O_2_ and H_2_O formation. Very recently, Mehta et al. implemented a core-polarity engineering strategy by designing two C_3_-symmetric pyridine-linked COFs that differ solely in the core unit (Fig. 7a): a lower-polarity benzene-cored Tab-Dfp and a higher-polarity triazine-cored Tta-Dfp [134]. XPS deconvolution reveals additional triazine-associated features in Tta-Dfp, including triazine-N (~ 398.4 eV) and C=N(triazine) (~ 285.7 eV) components (Fig. 7b), consistent with a more electron-deficient core; correspondingly, contact-angle measurements indicate a substantially more polar surface for Tta-Dfp (complete wetting, 0°) than for Tab-Dfp. Counter-intuitively, the less polar Tab-Dfp delivers markedly higher H_2_O_2_ selectivity (93.1% @ 0.4 V; n = 2.0; FE 90.5%) than Tta-Dfp (77.5% @ 0.3 V; n = 2.41; FE 66.9%), suggesting that higher polarity is not automatically synonymous with improved 2e⁻ selectivity. DFT calculations suggest that reducing core polarity weakens *OOH binding into a regime that suppresses O–O scission (disfavouring the 4e⁻ pathway) while facilitating H_2_O_2_ desorption along the 2e⁻ route. This mechanistic picture is supported by in situ electrochemical Raman spectroscopy, where the characteristic ~ 880 cm^−1^ band attributable to H_2_O_2_ formation maximises around 0.4 V (Fig. 7c), and by SECM mapping (Fig. 7d-f), which visualises stronger localised H_2_O_2_ signals on Tab-Dfp within the same potential window. Together with the previous case, this work again demonstrates that optimal polarisation strength must be matched to the dominant functional descriptor, here Δ*G*(*OOH) governing ORR selectivity, rather than pursued as a monotonic target.

Motivated by these non-monotonic trends, orientation-only inversion offers a straightforward approach to isolating polarity effects from other coupled structural and electronic variables. He et al. designed two isomeric imine-linked COFs (PyAl-TpbAm-COF and PyAm-TpbAl-COF, Fig. 7g) that differ solely in the orientation of the C=N bond, while maintaining nearly identical crystallinity, porosity, and optical gaps [114]. However, reversing the imine orientation from the amine-rich PyAm-TpbAl-COF to the aldehyde-rich PyAl-TpbAm-COF yields a ~ 3 × enhancement (from 0.7 to 2.1 mmol g^−1^ h^−1^) in photocatalytic hydrogen evolution. Temperature-dependent PL analysis indicates a pronounced reduction in E_b_ from 73.8 to 57.0 meV, accompanied by weakened delayed-PL intensity, consistent with more efficient exciton dissociation in PyAl-TpbAm-COF, along with higher photocurrent density, smaller EIS semicircles, and stronger TEMPO-EPR quenching. DFT links the orientation switch to enhanced local charge polarisation and delocalisation, as evidenced by the DFT-derived dipole moments and Sr/EDI values (Fig. 7h).

In addition to orientation-only inversion as a minimal causal perturbation, controlled defect engineering provides a complementary strategy to amplify polarisation in spatially confined regions in COFs. Hou’s group introduced localised donor-capped defects into a vinylene-linked COF (TtBda) via a one-step end-capping strategy, thereby generating embedded D–A motifs without globally disrupting lattice order, as shown in Fig. 7i [119]. At an optimised defect loading of 20%, the frameworks preserve crystallinity and permanent porosity, while the ground-state dipole moment increases systematically from 1.22 Debye (pristine) to 1.60, 1.91, and 3.01. Debye for the pyrene-, ferrocene-, and DpaB-modified variants, respectively. This enhancement is consistent with ESP maps and dielectric measurements showing strengthened local potential gradients (Fig. 7j). Correspondingly, *E*_*b*_ decreases markedly (Fig. 7k), accompanied by enhanced photocurrent response (Fig. 7l), reduced charge-transfer resistance (Fig. 7m), quenched steady-state PL (Fig. 7n), and shortened fluorescence lifetimes (Fig. 7o), collectively indicating accelerated exciton dissociation and charge-transport kinetics. Among these modified frameworks, the pyrene-modified analogue exhibits the most efficient charge transport, reflecting the role of rigid, highly conjugated donor plane in promoting *π*–*π* overlap and through-framework delocalisation. Furthermore, these local “dipolar boosters” yield a 7.8-fold increase in the apparent rate constant for water/oxygen-fuelled PET-RAFT photopolymerisation, achieving ~ 80% monomer conversion within 30 min.

In parallel, side-chain functionalisation provides another route to couple polarisation tuning with interfacial microenvironment control, which is often decisive in liquid-phase adsorption-photoreduction and mass-transfer-limited catalysis. In this regard, Qiu and co-workers started from BPDA-COF and progressively increased local polarisation by introducing –OH groups (DHBD-COF), followed by grafting electronegative PEO side chains via –OH-to-PEO conversion to form PEO-COF [124]. Although the crystallinity and porosity are preserved, side-chain modification drastically increases the molecular dipole (4.63 Debye vs. 0.93 Debye) and simultaneously reshapes the interfacial environment. As a result, PEO-COF enables rapid U(VI) photoreduction with a capacity of 1427.9 mg g^−1^, arising from polarisation-driven separation and PEO-enabled superhydrophilicity that enhances mass transfer. A more systematic substituent-series approach further demonstrates that local dipoles can be continuously graded within a fixed D–A scaffold to co-engineer band structure, exciton physics, and redox selectivity. Zhong et al. designed a family of *β*-ketoenamine D–A COFs (TpBD-X, X = –H, –OH, –NH_2_, –OCH_3_, –NO_2_, –SO_3_H), in which polar substituents on the benzidine unit finely modulate the local dipole field without altering the backbone topology [115]. Increasing substituent polarity progressively strengthens the internal electric field, narrows the bandgap, shifts band edges to more favourable positions for UO_2_^2+^ reduction, and markedly lowers E_b_ from ~ 105 to ~ 23–60 meV, enabling substantially improved U(VI) extraction kinetics and efficiencies. DFT analysis indicates that polar groups simultaneously lower the adsorption energy of UO_2_^2+^ and open multiple electron-transfer pathways, thereby linking substituent-level polarisation directly to interfacial reaction selectivity.

### Symmetry and Topology Control

In crystalline COFs, framework symmetry and lattice topology jointly determine how local dipoles combine at the collective level. While symmetry dictates whether dipoles are allowed to align or must cancel, topology governs how these dipoles are spatially connected, propagated, and periodically repeated along the framework skeleton and pore network. Together, these factors determine whether local dipoles coherently sum to a macroscopic polarisation field or, by symmetry, cancel into nonpolar multipolar states that lack a net internal field but exhibit distinct charge-transport characteristics. In frameworks where both symmetry and topology permit directional dipole propagation, deliberate dipole alignment can create framework-scale ESP gradients that directly drive directional carrier separation. By contrast, when lattice topology enforces alternating or closed-loop dipole arrangements, symmetry-enforced cancellation leads to multipolar lattices in which polarisation is locally retained but globally neutralised. In such cases, charge separation is not driven by a macroscopic internal field but instead facilitated through topology-enabled *π*-delocalisation and intraframework charge transfer. In this context, symmetry control should be viewed in concert with topological design as a counterintuitive approach to polarisation engineering. Rather than maximising a net dipole moment, arranging D–A motifs into symmetry-enforced multipolar lattices can suppress environment-induced polarisation perturbation while promoting extended *π*-delocalisation and intraframework charge transfer. This combination reduces exciton binding energies and suppresses recombination, thereby facilitating effective charge separation without relying on a strong internal electric field.

**Fig. 8:**
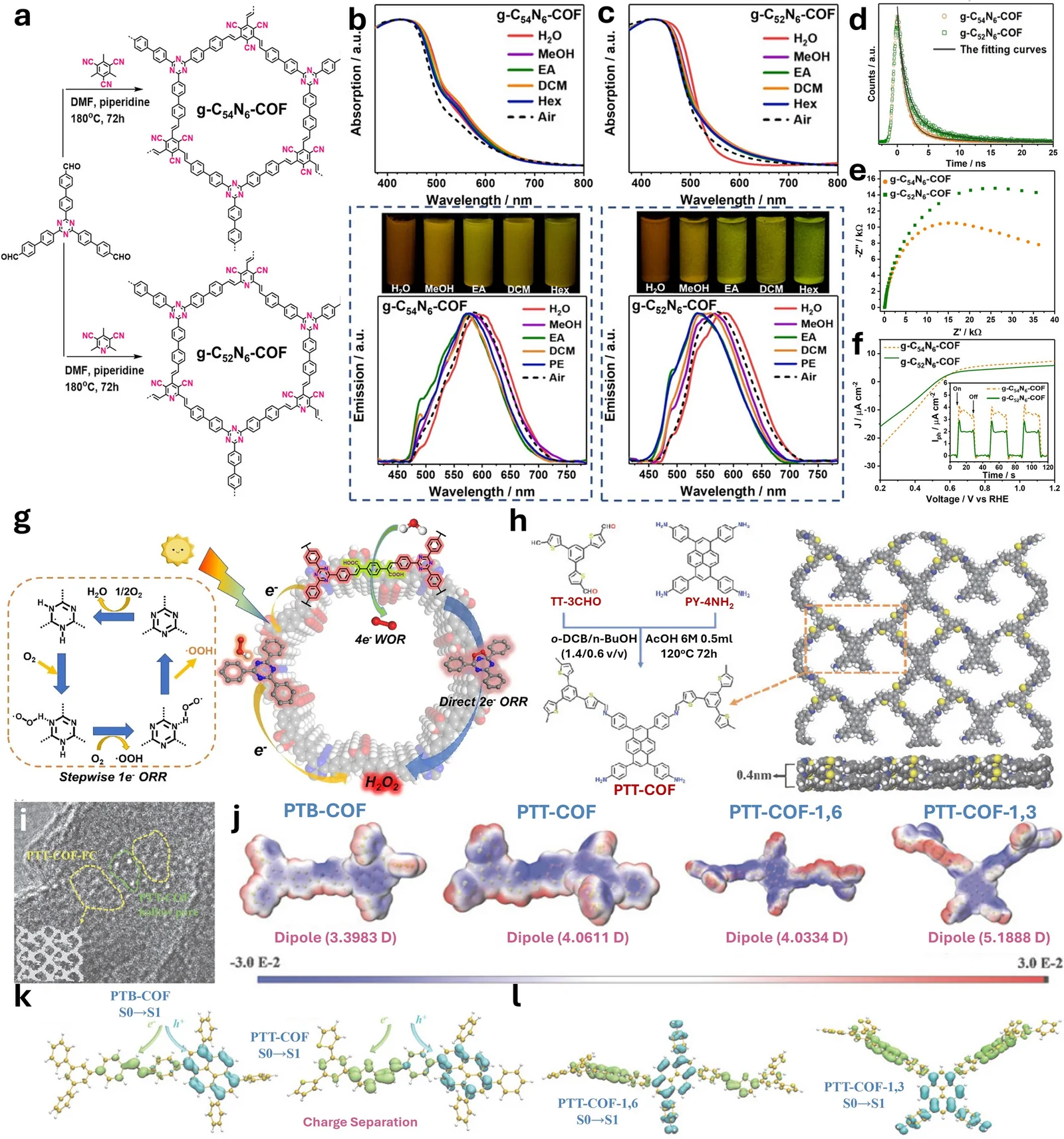
**a** Construction of g-C_54_N_6_–COF and g-C_52_N_6_–COF. **b** Absorption spectra in solvents of increasing polarity. **c** Emission spectra under 365-nm excitation in solvents of different polarity (0.5 mg in 20 mL solvent). **d** Time-resolved photoluminescence decay profiles. **e** Electrochemical impedance spectra recorded in the dark. **f** Linear sweep voltammograms and chopped photocurrent responses (inset) under visible light (≥ 420 nm) at 0.8 V versus RHE. Reproduced with permission [120]. Copyright 2020, Wiley. **g** Proposed reaction pathway for H_2_O_2_ photosynthesis over carboxylated TACOF. Reproduced with permission [121]. Copyright 2024, Wiley. **h** Synthesis and structural model of the thiophene-based substoichiometric COF, illustrating the non-centrosymmetric framework; right: top and side views of the AA-stacked lattice derived from PXRD refinement and modelling. **i** HRTEM image of the ferrocene-modified framework, showing preserved pore structure after post-synthetic functionalisation. **j** Ground-state charge-density distribution of centrosymmetric and non-centrosymmetric framework models, highlighting topology-induced charge polarisation (blue/red: electron accumulation/depletion). **k, l** Excited-state electron–hole distributions of representative symmetric and non-centrosymmetric connectivities, revealing topology-dependent charge separation. Reproduced with permission [122]. Copyright 2024, Elsevier

Xu and co-workers demonstrated the symmetry-tuned polarity using two vinylene-linked COFs constructed from monomers of different point-group symmetry, as shown in Fig. 8a [120]. Reticulating two D_3_h-symmetric building blocks led to g-C_54_N_6_–COF, featuring periodically aligned octupolar subunits that are topologically propagated throughout the framework, giving rise to a lattice-scale multipolar electronic state. Whereas replacing the D_3_h tricyanomesitylene with a C_2_v-symmetric 3,5-dicyanopyridine derivative yielded the less symmetric g-C_52_N_6_–COF, exhibiting a more dipolar electronic character. This symmetry imprint is directly reflected in their polarity readouts, in which g-C_52_N_6_–COF exhibits pronounced solvatochromism in both absorption and emission (Fig. 8b), whereas g-C_54_N_6_–COF shows only weak spectral shifts across solvents of increasing polarity (Fig. 8c), consistent with an octupolar/multipolar conjugated state that is comparatively insensitive to environmental polarisation. More importantly, the octupolar framework displays a series of charge-separation advantages, including a shorter PL lifetime (1.57 vs. 2.45 ns, Fig. 8d), a reduced E_b_ (42 vs. 86 meV), a smaller interfacial charge-transfer resistance (Fig. 8e), and an enhanced photocurrent response (23 vs. 16 μA cm^−2^ at 0.2 V vs. RHE; Fig. 8f). As expected, g-C_54_N_6_–COF enables visible-light-driven water-splitting half-reactions, delivering H_2_ evolution (2518.9 μmol h^−1^ g^−1^) and O_2_ evolution (51.0 μmol h^−1^ g^−1^), both of which outperform those of the less symmetric counterpart. It underscores that higher polarity does not necessarily imply superior photoconversion; instead, multipolar symmetry, even without a large macroscopic dipole, can unlock delocalised electronic states and accelerate charge generation and separation.

Multipolar topologies can translate into catalytic advantages when lattice-level charge directionality is integrated with liquid-phase transport and interfacial proton-coupled electron transfer (PCET). Extending the topology–polarity relationship, Xi et al. embedded octupolar and quadrupolar motifs into fully conjugated *sp*^2^-carbon COFs (TACOFs) by combining electron-withdrawing triazine nodes with donor vertices, hence establishing an intraframework charge-transfer network that favours oxygen reduction at the pore corners [121]. Crucially, post-synthetic conversion of peripheral nitrile groups into –COOH simultaneously introduces protic polar sites, increases pore wettability, and enhances proton conduction, which directly mitigates mass-transfer constraints and enables PCET-assisted conversion of O_2_^−^ intermediates in pure water. Among the series, TACOF-1-COOH exhibits the most favourable interfacial kinetics, as evidenced by the lowest charge-transfer resistance and the strongest photocurrent response, resulting in a high H_2_O_2_ production rate of 3542 μmol g^−1^ h^−1^ with a solar-to-chemical efficiency of 0.55%. DFT investigation suggests that the multipolar lattice concentrates electron density towards triazine-rich corners to promote ORR, whereas –COOH groups facilitate proton shuttling to accelerate key PCET steps. As illustrated in Fig. 8g, framework topology biases electronic flow towards catalytically active pore corners within the framework, while carboxylation selectively engineers the aqueous microenvironment required to convert this directional charge flow into efficient overall H_2_O_2_ photosynthesis.

Beyond monomer-symmetry selection, non-centrosymmetric network topology can intrinsically impose framework-level polarisation by breaking inversion symmetry at the lattice scale. Bai et al. proposed a topology-induced polarisation strategy by constructing a 2D substoichiometric COF (PTT-COF, Fig. 8h) from a thiophene-enriched tritopic linker and a tetratopic pyrene node [122]. Unlike conventional hexagonal lattices, the ~ 149° internal angle of thiophene frustrates inversion-symmetric tiling and biases the 2D stitching pathway towards a heart-shaped, non-centrosymmetric pore topology (Fig. 8i). This collective symmetry breaking generates a framework-scale polarisation field, manifested as asymmetric charge redistribution and a built-in electrostatic potential gradient (Fig. 8j), which is absent in the centrosymmetric benzene-linked analogue. Upon photoexcitation, this built-in polarisation directly reshapes exciton behaviour: electron–hole distribution and charge-centroid analyses reveal pronounced spatial separation and increased centroid distance in the polar lattice (Fig. 8k), indicative of weakened Coulombic binding. By contrast, symmetry-restored connectivity suppresses this separation despite identical building units (Fig. 8l), confirming that lattice-level symmetry breaking governs the polarisation effect. Such topology-induced polarisation, arising solely from lattice-level symmetry breaking rather than molecular dipoles, provides an electronic basis for accelerated exciton dissociation and directional charge transport, which can be further leveraged by hole-transport functionalisation to enable efficient photocatalysis. Beyond static symmetry breaking, engineering non-centrosymmetry through molecular dipole alignment offers a dynamic route to manipulate polarisation. Liang and co-workers recently designed a series of *β*-ketoenamine-linked BT-COFs where the synergy of asymmetric benzothiadiazole units and in-plane polarised linkages induces robust lattice-scale dipoles [123]. This dipole amplification breaks inversion symmetry and produces a measurable piezoelectric response, as confirmed by second-harmonic generation and PFM measurements. Importantly, systematic dipole optimisation revealed a direct correlation between framework polarity and piezoelectric coefficient, establishing molecular dipole engineering as a complementary pathway to topology-induced polarisation for achieving long-range non-centrosymmetry.

### Post-Synthetic Modification

In addition to pre-programming polarity during covalent framework synthesis, post-synthetic modification (PSM) offers an alternative to reprogram polarisation on an already crystallised COF lattice, while preserving long-range order and pore architecture. Rather than altering framework topology, PSM directly reshapes the internal ESP by introducing polar or ionic elements onto a fixed scaffold. From a mechanistic viewpoint, post-synthetic polarisation in COFs can be broadly divided into two modes: (i) ionic polarisation, where tethered cationic centres and their counterions or zwitterionic motifs generate strong local electrostatic fields and dipole layers, and (ii) covalent polarisation, in which polar functionalities or discrete dipolar junctions are chemically grafted onto the backbone or pore surface to modulate charge distribution. A key advantage of PSM lies in its decoupling of framework construction from field programming, enabling a single, robust lattice to be modularly upgraded into more strongly polarised architectures through targeted chemical transformations, rather than re-synthesising the framework.

**Fig. 9:**
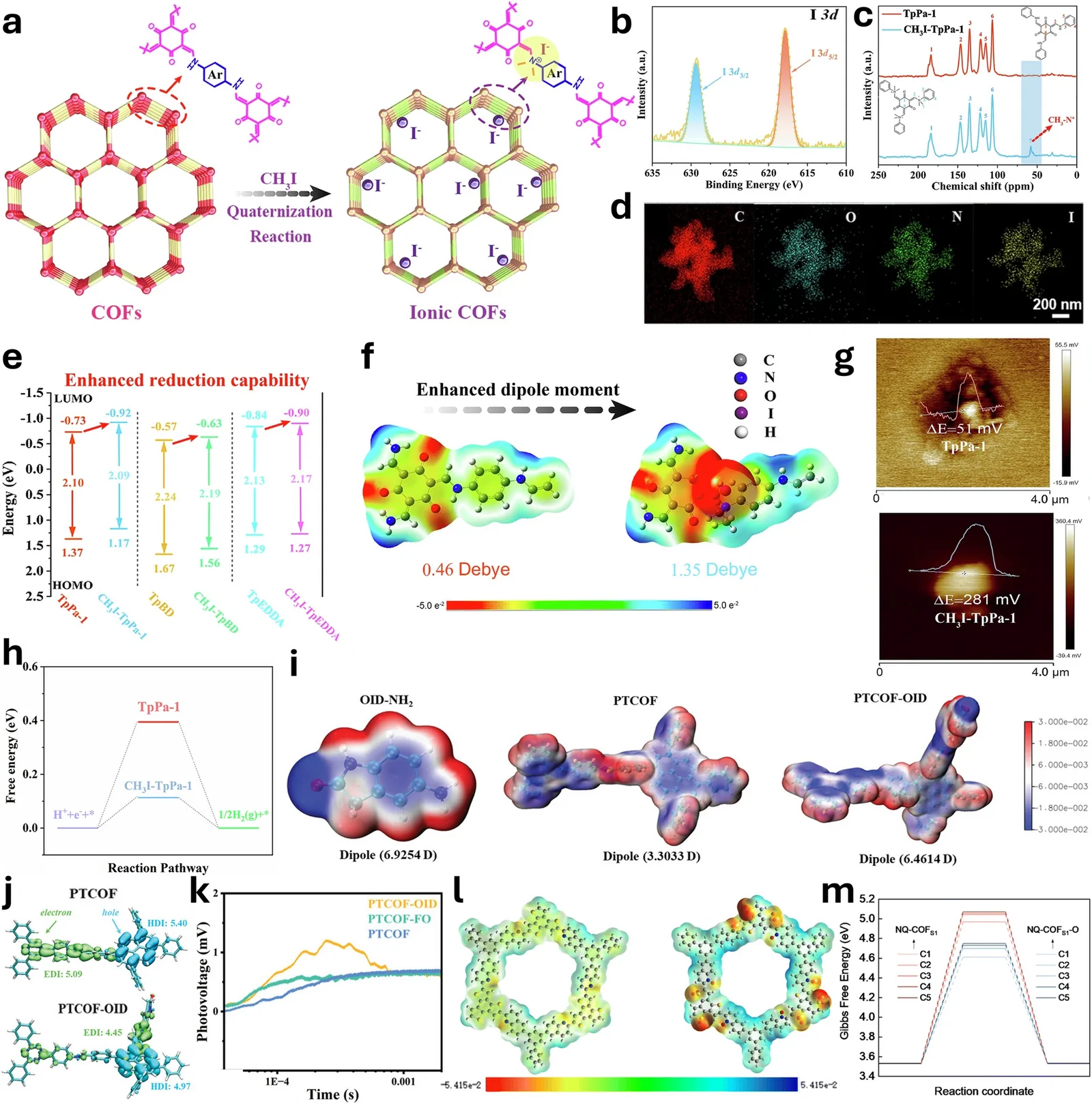
**a** Schematic illustration of post-synthetic quaternization of *β*-keto-enamine COFs to generate ionic frameworks. **b** High-resolution I 3*d* XPS spectrum of CH_3_I-TpPa-1. **c** Solid-state ^13^C CP/MAS NMR spectra of TpPa-1 and CH_3_I-TpPa-1. **d** EDX elemental mappings of C, N, O, and I, showing homogeneous iodide distribution. **e** Energy band alignment of pristine and quaternized *β*-keto-enamine COFs. **f** DFT-calculated ESP maps and corresponding dipole diagrams of TpPa-1 and CH_3_I-TpPa-1. **g** Surface-potential distributions measured by KPFM. **h** DFT-calculated free-energy diagrams for hydrogen evolution on TpPa-1 and CH_3_I-TpPa-1. Reproduced with permission [1]. Copyright 2024, Springer Nature. **i** Ground-state charge-density distributions of the pristine framework and the ketone-grafted analogue, where blue and red regions denote electron accumulation and depletion, respectively. **j** Real-space hole (blue) and electron (green) distributions in the excited state (isovalue = 0.0007), illustrating dipole-induced charge separation upon ketone grafting. **k** Transient surface photovoltage responses under 355-nm excitation, reflecting enhanced photoinduced carrier separation dynamics. Reproduced with permission [125]. Copyright 2023, Wiley. **l** ESP distributions of NQ-COFS1 and its oxidised analogue NQ-COFS1-O. **m** Gibbs free-energy profiles of the oxygen reduction reaction at representative active sites on NQ-COFS1 and NQ-COFS1-O, calculated at an equilibrium potential of 0.7V. Reproduced with permission [67]. Copyright 2025, RSC

Post-synthetic quaternization offers a prototypical ionic-pair strategy to program polarisation after crystallisation, creating spatially separated charge centres and a stable internal field without rebuilding the COF lattice. In our last work, we pioneered this concept in *β*-keto-enamine COFs (TpPa-1, TpBD, and TpEDDA) via CH_3_I treatment, generating tethered N^+^–CH_3_ frameworks paired with I^−^ counter-anions while retaining crystallinity, morphology, and microporosity (Fig. 9a) [1]. Successful ion-pair installation is confirmed by the emergence of C–N^+^/I 3*d* features in XPS (Fig. 9b), the N^+^–CH_3_ resonance at ~ 57 ppm in solid-state ^13^C NMR (Fig. 9c), and homogeneous iodide distribution in EDS mapping (Fig. 9d). Mechanistically, ionic polarisation arises from the enforced separation of the cationic skeleton and iodide anions, which redistributes electron density and establishes a lattice-confined electrostatic potential gradient. More importantly, quaternization systematically deepens the LUMO levels (to − 0.92/ − 0.63/ − 0.90 eV for CH_3_I-modified TpPa-1/TpBD/TpEDDA), consistent with electrostatic stabilisation of π* states in the vicinity of N^+^ centres (Fig. 9e). DFT-ESP analysis further shows an increased dipole moment from 0.46 to 1.35 Debye (Fig. 9f), while KPFM confirms this field amplification with the surface potential rising from 51 to 281 mV, corresponding to an ~ 6.4 × stronger BIEF directed from the Tp unit towards I^−^ (Fig. 9g). Crucially, this polarisation reduces *E*_b_ (from 75.3 to 63.9 meV) and enhances charge separation and transfer, as evidenced by a series of photoelectrochemical and spectroscopic measurements. Also, iodide plays a dual interfacial role as an electron-extracting moiety and an energetically favourable H* adsorption site (Δ*G*_H*_ ≈ 0.12 eV vs. ≈ 0.40 eV on O sites in the pristine framework, Fig. 9h), rationalising Pt-free hydrogen evolution. Consequently, CH_3_I-TpPa-1 delivers a 42-fold enhanced H_2_ evolution rate (9.21 vs. 0.22 mmol g^−1^ h^−1^), along with high recyclability (4 cycles/20h), seawater tolerance, and wavelength-dependent apparent quantum efficiency.

Neutral grafting of polar small molecules provides an orthogonal PSM strategy to install covalent dipolar junctions within COF pores, enabling local polarisation enhancement without introducing mobile counterions. Huang et al. realised this concept in a 2D substoichiometric framework (PTCOF) by exploiting periodically uncondensed aldehyde groups as reactive handles to covalently anchor polar ketone molecules (OID) via Schiff-base condensation [125]. Importantly, this post-modification preserves the parent backbone topology while embedding molecular dipoles periodically along the pore walls. At the ground-state level, DFT calculations reveal an increase in intrinsic polarity, where the introduction of OID elevates the dipole moment from 3.30 Debye in pristine PTCOF to 6.46 Debye in PTCOF-OID (Fig. 9i), confirming the successful imprinting of a stronger electrostatic asymmetry into the *π*-conjugated framework. This enhanced polarity also reshapes excited-state charge behaviour. Real-space electron–hole distribution analysis shows that ketone junctions drive spatial separation of photoexcited carriers, with electrons preferentially localised towards the grafted dipolar units while holes remain on the framework backbone (Fig. 9j), indicating weakened Coulombic binding and facilitated exciton dissociation. The impact of this dipolar junction on charge-separation dynamics is further corroborated experimentally. Transient surface photovoltage measurements display a markedly enhanced positive signal for PTCOF-OID relative to the pristine framework (Fig. 9k), reflecting more efficient generation and extraction of free carriers under photoexcitation. As a direct functional consequence of this polarisation-enabled separation, PTCOF-OID achieves a H_2_ evolution rate of 29.29 mmol g^−1^ h^−1^, corresponding to a 5.6-fold enhancement over the unmodified scaffold.

Defect-generating PSM has also been explored as a means to reconstruct local electric fields and adsorption microenvironments. In a recent example, He et al. converted peripheral nitrile groups in a *β*-ketoenamine COF into carboxylate defects, generating defect-rich frameworks in which an intermediate defect density maximises dipole strength while preserving structural integrity [126]. Photophysical and electrochemical measurements indicate that carboxyl defects enhance internal-field-assisted charge separation, while Debye–Hückel analysis suggests a spatially extended electrostatic field that promotes interfacial UO_2_^2+^ enrichment. As a result, the optimised material exhibits high U(VI) uptake and enables efficient photoreduction to U(IV). Although defect-induced polarisation lacks the structural precision of covalent dipole grafting, such approaches demonstrate that polarisation engineering and interfacial enrichment can be co-induced through defect chemistry, offering a pragmatic strategy for reactions governed by coupled charge separation and mass transfer.

Oxidation-enabled PSM provides a concise route to generate zwitterionic/ionic motifs in situ, simultaneously amplifying framework polarisation and reshaping reaction-pathway selectivity. Pang and co-workers reported a mild post-oxidation strategy for a thiophene/quinoline-linked COF (NQ-COFS1), in which m-chloroperbenzoic acid converts the parent framework into its oxidised analogue while preserving the hcb topology, nanotube morphology, and crystallinity [67]. The introduction of N^+^–O^−^ and sulfone (–SO_2_–) functionalities induces a highly asymmetric electrostatic potential distribution (Fig. 9l), nearly tripling the ground-state dipole moment from 1.30 to 3.93 Debye, accompanied by bandgap narrowing (from 2.19 to 1.85 eV) and band-edge shifts that thermodynamically favour the two-electron oxygen reduction pathway. This strengthened polarisation lowers *E*_*b*_ and accelerates charge separation and transport. More importantly, the oxidised sulfone/N^+^–O^−^ motifs selectively stabilise the OOH intermediate, as revealed by DFT free-energy analysis (Fig. 9m), thus biasing the ORR towards the 2e⁻ H_2_O_2_ pathway rather than merely enhancing overall activity. As a result, the oxidised framework delivers an H_2_O_2_ production rate of 6.07 mmol g^−1^ h^−1^ with an apparent quantum efficiency of 9.2% at 420 nm, which is almost 95-fold higher than that of the pristine counterpart.

## Conclusions and Outlook

Based on the above discussion, it is clear that polarisation engineering has emerged as a powerful and unifying principle for COF catalysis, not simply as a means to enhance charge separation, but as a route to deliberately programme internal electrostatics across multiple length scales. In this review, we have comprehensively summarised the multiscale origins of polarisation in COFs from a hierarchy of design elements, spanning bond-level dipoles, D–A asymmetry along the *π*-skeleton, framework symmetry and topology, stacking modes, defects, and ion-pair motifs. On this basis, we organised a practical set of strategies to introduce, strengthen, or reorient polarisation for various photocatalytic and electrocatalytic systems. Across these distinct strategies, a recurring mechanistic picture emerges: polarisation reshapes the electrostatic potential landscape and built-in fields, reduces effective exciton binding, facilitates charge separation, and directs carriers towards reactive interfaces, where adsorption geometries and reaction barriers are further modulated. Importantly, polarisation should not be viewed as a monotonic stronger-is-better parameter. Rather, an optimal polarisation window is likely to exist for different catalytic reactions, determined by the specific energetic and kinetic descriptors governing each system. In photocatalytic COFs, the built-in electric field should be sufficiently strong to meaningfully reduce exciton binding energy and facilitate charge separation, yet not so strong as to induce excessive band bending or perturb frontier orbital alignment. In electrocatalytic systems governed by Sabatier-type relationships, polarisation-induced modulation of adsorption free energies should move key intermediates towards thermodynamic optimality without over-stabilising them. In piezocatalytic systems, the effective window is additionally constrained by dielectric screening and surface charge accumulation under electrolyte conditions. Therefore, effective polarisation engineering requires quantitative matching of field strength and spatial distribution to the specific reaction coordinate and interfacial dielectric environment. Optimal performance is inherently reaction- and interface-specific, requiring both the magnitude and spatial distribution of polarisation to be matched to the dominant kinetic or thermodynamic bottleneck, whether exciton dissociation, interfacial charge transfer, intermediate stabilisation, or product desorption, and to the selectivity descriptor governing competing multi-electron pathways. These distinct yet interconnected roles of polarisation in COF catalysis are summarised in Table 2.

**Table 2:** Role of polarisation in linking electronic structure and catalytic function in COFs

Target reaction	Dominant bottleneck	Key parameter	Role of polarisation	Representative strategies	References
Photocatalytic H_2_ evolution (HER)	Low free-carrier yield due to strong excitonic effects	*E*_*b*_; carrier lifetime (*τ*)	Lower *E*_*b*_ and promote spatial e–h separation to increase usable electrons	Linkage polarity tuning; D–A frameworks; heteroatom substitution; ionic PSM	[63, 76, 109, 110, 112, 113]
Overall water splitting/half reactions	Inefficient directional delivery of electrons and holes	Real-space band-edge separation; internal field direction	Drive electrons and holes towards spatially separated redox sites	Linkage conversion; D–A or A1–D–A2 design; symmetry/topology control	[109, 110, 120, 122, 123]
Selective H_2_O_2_ production (2e^−^ ORR)	Competition between 2e^−^ and 4e^−^ pathways	Δ*G*(*OOH) relative to *O/*OH; electron-transfer number (*n*)	Moderately tune electrostatic stabilisation of *OOH to suppress O–O cleavage	Controlled polarity grading; oxidation-induced ionic motifs; D–A coupling	[55, 67, 134]
Photocatalytic pollutant degradation/ROS chemistry	Slow interfacial charge transfer and O_2_ activation	Surface potential; interfacial *R*_ct_	Enhance surface electric fields to facilitate charge injection and ROS formation	Zwitterionic linkages; heterocycle locking; D–A architectures	[76, 111, 116–118]
Ion-coupled photocatalysis (e.g., U(VI) reduction)	Decoupling of ion adsorption and electron delivery	Ion adsorption capacity; local electron flux	Couple electrostatic ion enrichment with internal charge separation	Polar side chains; functional group conversion; defect-induced fields	[115, 124, 126]
Aqueous/scalable catalytic systems	Screening of internal fields by solvent and electrolytes	Effective field range; wettability	Maintain polarisation effects under dielectric screening	Ionic polarisation; hydrophilic polar groups; PSM	[1, 124, 126]

Despite rapid progress, translating polarisation into a predictive, transferable catalytic design still remains challenging. First and foremost, the central difficulty lies in disentangling polarisation effects from correlated structural and chemical variables, including crystallinity, defect density, wettability, and the nature of active sites, which often evolve simultaneously. Secondly, polarisation metrics are not yet unified across studies. Ground-state descriptors such as dipole moments or ESP contrast, and field-level probes including KPFM, UPS, or SPV, are typically measured under idealised conditions, whereas catalysis proceeds in environments dominated by electrolyte screening, solvent polarisation, and dynamic surface charging that can substantially renormalise internal fields. Additionally, another complication is the intrinsic spatial heterogeneity of polarisation in COFs, which can vary across domains, grain boundaries, and pore-surface regions, and may further couple to ion migration or chemical reconstruction under operating conditions. Resolving these issues will require closer integration of quantitative descriptors, operando characterisation, and theoretical investigations that explicitly incorporate realistic interfaces and electrochemical environments.

Looking forward, several directions are promising for moving polarised COFs from compelling case studies towards a broadly catalytic field. A priority is establishing polarisation descriptors that are both comparable across systems and relevant under operating conditions. This will require clearer links between ground-state polarity metrics (dipole moments, ESP contrast) and the effective internal fields that persist under electrolyte screening, along with excitonic and kinetic readouts (e.g., *E*_b_, TRPL, or TAS components, photocurrent responses, EIS-derived *R*_ct_, etc.) measured under matched conditions. In this context, operando techniques capable of resolving local potentials and charge accumulation, alongside in situ identification of key intermediates, will be critical for validating polarisation-directed pathway control rather than post-correlations. Beyond quantification, future design strategies should move past the simple notion of stronger dipoles towards deliberate spatial programming of polarisation. Anisotropic fields, potential gradients, and multipolar architectures offer opportunities to channel electrons and holes towards distinct interfaces, while concomitant engineering of pore microenvironments will be necessary to ensure that these internal fields remain operative under aqueous and electrochemical screening. Achieving such control will demand closer integration between molecular design, framework topology, and interfacial chemistry. In addition to static built-in fields, an intriguing longer-term direction is the exploration of switchable or stimulus-responsive polarisation in COFs. Switchable polarisation, if achievable, could in principle enable reversible modulation of adsorption energetics, band alignment, and interfacial charge distribution, thereby offering dynamic control over reaction pathways and electrochemical interfaces beyond static field programming. While ferroelectricity has been demonstrated in certain organic molecular crystals and polymeric systems, most current systems are non-ferroelectric, as their polarisation predominantly arises from fixed electronic asymmetry rather than bistable lattice distortions. Conceptual routes towards tunable electrostatic landscapes may emerge through the incorporation of conformationally dynamic dipolar units, modulation of stacking registry in layered COFs, or reversible ionic/guest reconfiguration under external stimuli [135]. Realising such behaviour would require rigorous differentiation between genuine polarisation switching and artefacts associated with electrostatic charging or ionic migration, extending polarisation engineering beyond static field programming towards dynamic control of reaction environments.

Progress will also hinge on mechanism-aware computation across length scales. Explicit treatment of solvent and electrolyte effects, field-coupled adsorption and PCET kinetics, and multiscale models that bridge periodic frameworks with realistic surfaces and defects are needed to translate polarisation descriptors into predictive guidance for selectivity and stability. In parallel, data-driven approaches may accelerate the identification of “just-right” polarisation regimes tailored to specific reactions. Last but not least, the functional applications of polarisation engineering should continue to expand beyond activity enhancement. Polarisation is uniquely positioned to act as an intrinsic selector in multi-electron reactions where intermediate binding and desorption compete, as already exemplified by oxygen reduction, which can be extended to other applications such as CO_2_ conversion, nitrogen fixation, biomass valorisation, and pollutant transformation. Realising this potential will require scalable processing of polarised COFs into electrodes, membranes, and flow-compatible architectures, enabling polarisation-enabled kinetics and selectivity to be expressed under practical, mass-transport-limited conditions. Beyond catalysis, the conceptual framework of polarisation engineering in COFs is increasingly extending to electrochemical energy storage [136]. In these systems, programmed internal electrostatics can regulate ion adsorption energetics and local solvation structures, suppress undesired anion or polysulfide migration through combined electrostatic interactions and structural confinement, and establish directional ion-transport pathways, thereby mitigating concentration polarisation and stabilising dynamic electrode–electrolyte interphases under high-rate or high-temperature operation. Recent examples include polarised-site COFs that confine polysulfide intermediates to alleviate the shuttle effect in Li–S chemistry [137], polarisation-regulated COF architectures that improve ionic selectivity and transport in composite electrolytes for solid-state Li-metal batteries [138], and related COF-enabled strategies where electrostatic interactions are leveraged to couple ion management with interfacial stability under practical cycling conditions [139].

By linking the origins of polarisation to a range of engineering strategies, quantitative descriptors, and electrochemical functionalities, this review shows that internal electrostatics in COFs can be deliberately programmed to jointly regulate charge dynamics, ion transport, and interfacial reactions. As polarisation becomes more standardised, operando validation becomes more accessible, and spatial control becomes more deliberate, polarisation engineering is expected to evolve from a descriptive concept into a predictive design principle for COF catalysts, enabling rational tuning of activity, selectivity, and long-term durability.
